# Inhibiting the NADase CD38 improves cytomegalovirus-specific CD8^+^ T cell functionality and metabolism

**DOI:** 10.1172/JCI179561

**Published:** 2024-07-02

**Authors:** Nils Mülling, Felix M. Behr, Graham A. Heieis, Kristina Boss, Suzanne van Duikeren, Floortje J. van Haften, Iris N. Pardieck, Esmé T.I. van der Gracht, Ward Vleeshouwers, Tetje C. van der Sluis, J. Fréderique de Graaf, Dominique M.B. Veerkamp, Kees L.M.C. Franken, Xin Lei, Lukas van de Sand, Sjoerd H. van der Burg, Marij J.P. Welters, Sebastiaan Heidt, Wesley Huisman, Simon P. Jochems, Martin Giera, Oliver Witzke, Aiko P.J. de Vries, Andreas Kribben, Bart Everts, Benjamin Wilde, Ramon Arens

**Affiliations:** 1 Department of Immunology, Leiden University Medical Center, Leiden, The Netherlands.; 2 Department of Nephrology, University Hospital Essen, University Duisburg-Essen, Essen, Germany.; 3 Department of Parasitology, Leiden University Centre for Infectious Diseases, Leiden University Medical Center, Leiden, The Netherlands.; 4 Department of Infectious Diseases, University Hospital Essen, University Duisburg-Essen, Essen, Germany.; 5 Department of Medical Oncology,; 6 Center for Proteomics and Metabolomics, and; 7 Department of Internal Medicine, Leiden University Medical Center, Leiden, The Netherlands.

**Keywords:** Immunology, Transplantation, Glucose metabolism, T cells

## Abstract

Cytomegalovirus (CMV) is one of the most common and relevant opportunistic pathogens in people who are immunocompromised, such as kidney transplant recipients (KTRs). The exact mechanisms underlying the disability of cytotoxic T cells to provide sufficient protection against CMV in people who are immunosuppressed have not been identified yet. Here, we performed in-depth metabolic profiling of CMV-specific CD8^+^ T cells in patients who are immunocompromised and show the development of metabolic dysregulation at the transcriptional, protein, and functional level of CMV-specific CD8^+^ T cells in KTRs with noncontrolled CMV infection. These dysregulations comprise impaired glycolysis and increased mitochondrial stress, which is associated with an intensified expression of the nicotinamide adenine dinucleotide nucleotidase (NADase) CD38. Inhibiting NADase activity of CD38 reinvigorated the metabolism and improved cytokine production of CMV-specific CD8^+^ T cells. These findings were corroborated in a mouse model of CMV infection under conditions of immunosuppression. Thus, dysregulated metabolic states of CD8^+^ T cells could be targeted by inhibiting CD38 to reverse hyporesponsiveness in individuals who fail to control chronic viral infection.

## Abstract 2

Kidney transplant recipients who are unable to control cytomegalovirus infection have dysfunctional CD8^+^ T cells that can be metabolically reinvigorated by targeting the NADase CD38.

## Graphical abstract

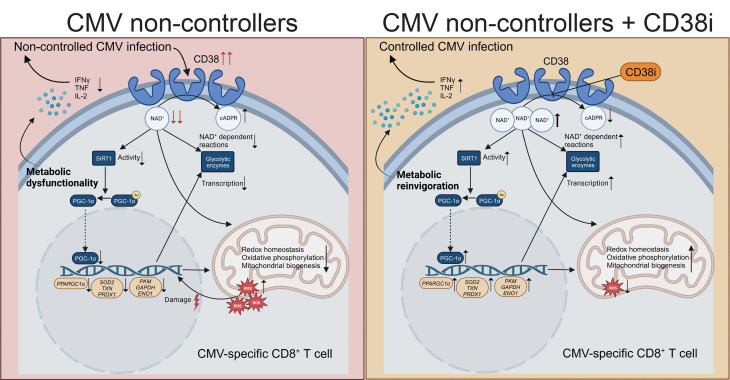

## Introduction

Human cytomegalovirus (CMV) is one of the most encountered opportunistic viral pathogens in kidney transplant recipients (KTRs) (1). Although major advances in the treatment and prophylaxis of CMV-associated complications have been achieved in the last decades, CMV still remains an important cause of morbidity and mortality in KTRs. The clinical course of CMV infections in KTRs range from asymptomatic reactivation to invasive disease with organ involvement such as colitis, hepatitis, retinitis, or pneumonia (1). CMV infection may also have detrimental indirect effects, including an increased risk of graft rejection or infections with other pathogens, even during asymptomatic CMV reactivation (2, 3). To stratify the risk of CMV infection after kidney transplantation, clinicians evaluate the anti-CMV IgG serology match of the donor (D) and recipient (R) (4), where “+” indicates that the individual is anti-CMV IgG positive and “–” indicates that the individual is anti-CMV IgG negative. High-risk patients (D+/R–) receive prophylaxis with antiviral drugs such as valganciclovir; intermediate-risk patients (D±/R+) receive either prophylaxis or a preemptive strategy; and low-risk patients (D–/R–) do not receive prophylaxis (5, 6). Valganciclovir is used for prophylaxis and treatment of CMV infection, while intravenous ganciclovir is used for severe disease. However, side effects of these drugs are common, and especially myelotoxicity or emerging viral resistance are major limitations of prolonged usage (7). Cellular immunity is a crucial component in the defense against CMV, to which NK cells and especially CD8^+^ T cells are of utmost importance (8). In the immune competent host, CMV-specific CD8^+^ T cells remain functional and lack high expression of inhibitory receptors (9), while in individuals who are immunocompromised with uncontrolled infection these CMV-specific T cells are less functional. Nonetheless, these T cells are not characterized by excessive inhibitory receptor expression (10–13). Evidently, there is a need to identify strategies to improve CD8^+^ T cell function in KTRs to alleviate CMV-associated disease.

In recent years, high-dimensional techniques have improved the knowledge about the single-cell kinetics of immune subsets in the context of transplant patients with CMV infection (8, 14, 15). However, the aforementioned studies mainly focused on the phenotypical characterization of immune cells, whereas detailed insights into the interplay between effector functionality and metabolism of CMV-specific T cells in transplant patients are still lacking (16). Additionally, T cell metabolism has emerged as a promising target to functionally restore antigen-specific T cells in the fields of oncology and chronic viral infections (17, 18). Here, we performed detailed metabolic analysis of CMV-specific CD8^+^ T cells from KTRs with controlled and uncontrolled CMV infection. We report a dysregulated metabolic state of CMV-specific CD8^+^ T cells in KTRs with impaired control of CMV infection, which is linked to repressed cytokine responses. The metabolic alterations are associated with increased expression of the ectoenzyme CD38 on CMV-specific CD8^+^ T cells. Moreover, inhibition of the NADase activity of CD38 restored cellular metabolism and improved T cell function as evidenced by enhanced cytokine production. These results were confirmed in a controlled mouse infection model.

## Results

### Frequency and phenotype of CMV-specific CD8^+^ T cells are unaffected by loss of viral control while cytokine production is decreased.

**Figure 1:**
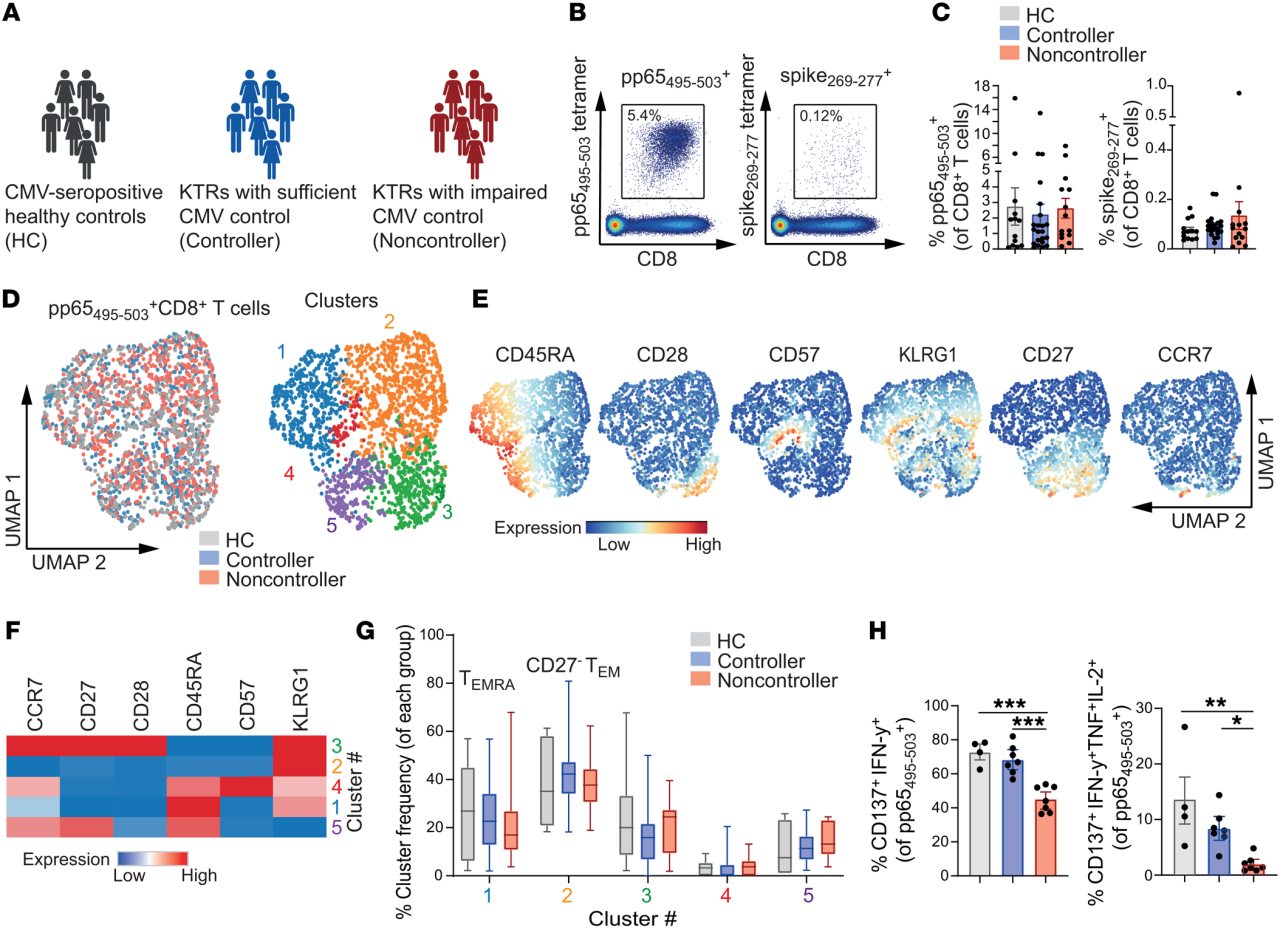
Frequency and phenotype of CMV-specific CD8^+^ T cells are unaffected by loss of viral control while cytokine production is decreased. (**A**) Graphical overview of main study groups. Created with biorender.com. (**B**–**G**) PBMCs of HCs (*n* = 13), controllers (*n* = 22), and noncontrollers (*n* = 14) were analyzed ex vivo. (**B**) Representative plots of pp65_495–503_ and spike_269–277_–specific CD8^+^ T cells in the same host. (**C**) Frequencies of pp65_495–503_ and spike_269–277_–specific CD8^+^ T cells of total CD8^+^ T cells. (**D**) UMAP analysis (left) and FlowSOM consensus metaclustering with 5 clusters (right) was performed on pp65_495–503_–specific CD8^+^ T cells (downsampled to equal numbers between groups). (**E**) Expression intensity of cell surface markers. (**F**) Hierarchically clustered heatmap of phenotypes of the 5 clusters shown in **D**. Marker expression per cluster as z score of median signal intensity per channel. (**G**) Cluster frequencies of pp65_495–503_–specific CD8^+^ T cells. (**H**) Percentage IFN-γ^+^CD137^+^ (left) and IFN-γ^+^TNF^+^IL-2^+^CD137^+^ cells (right) of pp65_495–503_–specific CD8^+^ T cells stimulated for 20 hours with peptide (*n* = 4–7/group). Data are presented as mean ± SEM or as boxplot. Bounds of the boxes indicate upper and lower quartile, lines indicate median, whiskers indicate min and max. Each dot represents an individual. Statistical analysis by 1-way ANOVA with Tukey’s test for multiple comparisons or 2-way ANOVA with Šidák’s test for multiple comparisons. *P < 0.05; **P < 0.01; ***P < 0.001; ****P < 0.0001.

CMV infection can cause disease in KTRs. To evaluate mechanisms underlying uncontrolled CMV infection, KTRs were subdivided in controllers — patients who are in immunological control of the virus — and noncontrollers — patients who develop severe, recurring CMV viremia with clinical symptoms (Figure 1A and Supplemental Tables 1 and 2; supplemental material available online with this article; https://doi.org/10.1172/JCI179561DS1). Peripheral blood of noncontrollers was sampled between 2 weeks and 10 weeks after latest CMV replication onset and was compared with blood of controllers as well as people who were CMV-seropositive healthy controls (HCs). CMV-specific CD8^+^ T cells were studied in-depth and evaluated along with SARS-CoV-2–specific CD8^+^ T cells, which served as an internal control virus-specific T cell population.

The frequency of CMV pp65_495–503_–specific CD8^+^ T cells within the total CD8^+^ T cell population was increased compared with SARS-CoV-2 spike_269–277_–specific CD8^+^ T cells within the same patients, which underlines the expansion of virus-specific T cells to CMV persistence in the host (Figure 1, B and C, and Supplemental Figure 1A). The frequencies of both virus-specific T cell populations, however, did not differ between HCs, controllers, and noncontrollers (Figure 1C), indicating that uncontrolled CMV infection does not necessarily lead to elevated T cell expansion in the peripheral blood. Moreover, phenotypical analysis by unsupervised Uniform Manifold Approximation and Projection (UMAP) dimensionality reduction and cluster analysis of the conventional memory differentiation phenotype of CMV-specific CD8^+^ T cells revealed the inflationary CCR7^–^CD27^–^CD28^–^KLRG1^+^ cells, either expressing or not expressing CD45RA, resembling effector-memory or terminally differentiated effector memory (T_EMRA_) subsets. However, no differences between HCs, controllers, and noncontrollers were observed (Figure 1, D–G), highlighting that neither the frequencies within the total CD8^+^ T cell pool nor the memory differentiation phenotypes distinguish KTR CMV controllers from noncontrollers. In contrast, comparative analysis of CMV and SARS-CoV-2–specific CD8^+^ T cells by UMAP dimensionality reduction did reveal distinct memory differentiation phenotypes between these viral-specific T cells. Whereas the pp65_495–503_–specific CD8^+^ T cells consisted mainly of the CCR7^–^CD27^–^CD28^–^ T_EM_/T_EMRA_ phenotype, the spike_269–277_–specific CD8^+^ T cells showed a T_EM_ phenotype characterized by high expression of CD27 and CD28 (19) (Supplemental Figure 1, C–F).

To examine the functional responsiveness of CMV-specific CD8^+^ T cells to cognate antigen, we stimulated the cells with pp65_495–503_ (NLVPMVATV) peptide and assessed cytokine production. Notably, we detected lower frequencies of IFN-γ^+^ and IFN-γ^+^TNF^+^IL-2^+^ (triple positive) CD8^+^ T cells in noncontrollers than in HCs and KTR CMV controllers (Figure 1H and Supplemental Figure 1B), indicating a link between a diminished functionality of CMV-specific CD8^+^ T cells and the clinical observation of noncontrolled CMV infection. In contrast, frequencies of IFN-γ^+^CD8^+^ T cells after stimulation with spike_269–277_ peptide did not differ between controllers and noncontrollers (Supplemental Figure 1G). Moreover, the blood values of the immunosuppressants tacrolimus and mycophenolic acid (MPA), which are routinely provided in KTRs to prevent graft rejection, and the percentage of FOXP3^+^ Tregs were not different between controllers and noncontrollers (Supplemental Figure 1, H and I).

### Transcriptional analysis of metabolic pathways reveals downregulation of genes related to glycolysis and mitochondrial respiration in CMV-specific CD8^+^ T cells of noncontrollers.

**Figure 2:**
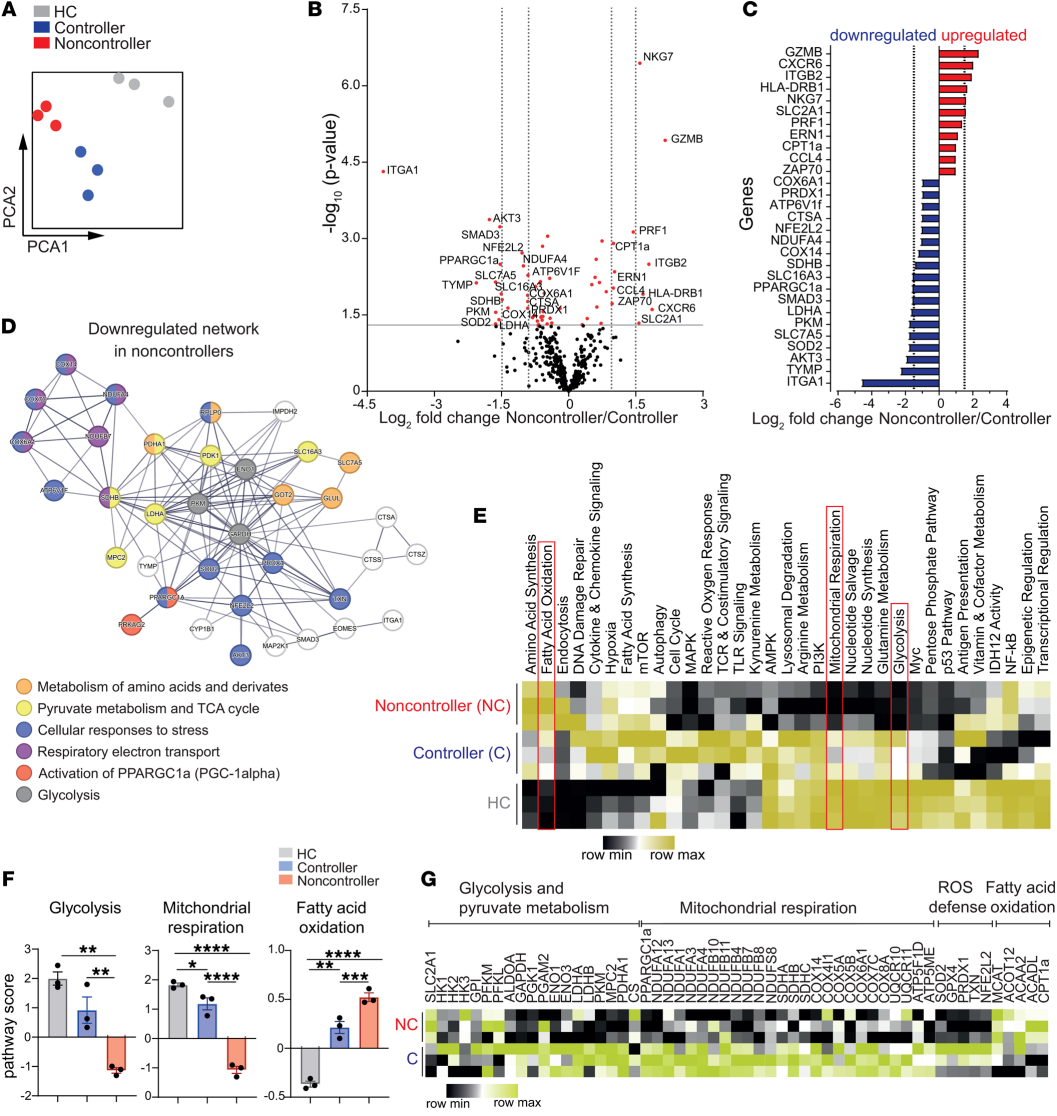
Transcriptional analysis of metabolic pathways reveals downregulation of genes related to glycolysis and mitochondrial respiration in CMV-specific CD8^+^ T cells of noncontrollers. pp65_495–503_–specific CD8^+^ T cells (*n* = 3 independent donors/group) were sorted from PBMCs, and gene expression was profiled using the Nanostring nCounter Metabolic Pathways Panel. (**A**) Principal component analysis (PCA) of all 768 analyzed genes. Each dot represents an individual.(**B**) Volcano plot showing differentially expressed genes between controllers and noncontrollers. Genes with significant differential expression (*P* < 0.05) are highlighted in red and of those, genes with an at least log_2_(1)-fold differential expression are indicated. (**C**) Bar plot of differentially expressed genes between controllers and noncontrollers. Upregulated genes in noncontrollers (compared with controllers) are colored red, downregulated genes in noncontrollers are colored blue. (**D**) STRING interaction network of proteins encoded by genes downregulated in noncontrollers compared with controllers (*P* < 0.05). Proteins with a strong confidence interaction score (> 0.7) are shown. (**E**) Heatmap of metabolic pathways. Scores are displayed as z score. Further analyzed pathways are highlighted in red. (**F**) Bar graphs of pathway scores. Scores are presented as relative expression. Each dot represents an individual. (**G**) Heatmap showing the expression of single genes related to glycolysis and pyruvate metabolism, mitochondrial respiration, ROS defense, and fatty acid oxidation in controllers and noncontrollers. Scores are displayed as z score. Data are presented as mean ± SEM. Statistical analysis by 1-way ANOVA with Tukey’s test for multiple comparisons. *P < 0.05; **P < 0.01; ***P < 0.001; ****P < 0.0001.

The functionality of CD8^+^ T cells is coupled to their metabolic state (17). To investigate the metabolism of CMV-specific CD8^+^ T cells, pp65_495–503_–specific CD8^+^ T cells of HCs, KTR CMV controllers, and noncontrollers were sorted (Supplemental Figure 2A) and expression of 768 genes involved in cellular metabolism was assessed using multiplexed hybridization technology (Nanostring nCounter Max platform). Principal component analysis (PCA) based on all analyzed genes showed a clear segregation of the 3 groups (Figure 2A). Differential expression (DE) analysis revealed 60 genes that were distinctly expressed between controllers and noncontrollers (Figure 2, B–D). Among the most upregulated genes in noncontrollers, we found an increased expression of *GZMB*, *NKG7*, *PRF1*, *HLA-DRB1*, and *ZAP70*, indicating an enhanced state of TCR-mediated activation (Figure 2, B and C). We also detected an increased expression of the gene encoding the main glucose transporter GLUT1 (*SLC2A1*) in CMV pp65_495–503_–specific CD8^+^ T cells of noncontrollers. Remarkably, transcripts of genes encoding crucial enzymes in glycolysis such as *LDHA*, encoding for lactate dehydrogenase A, and *PKM*, encoding for pyruvate kinase, were downregulated in noncontrollers. Corresponding with this downregulation, the gene expression of enzymes that link glycolysis with the tricarboxylic acid (TCA) cycle such as *MPC2* and *PDHA1* were decreased in noncontrollers. However, genes encoding components of the TCA cycle, such as citrate synthase (*CS*) did not show differential expression. In addition to downmodulation of glycolysis-related genes, noncontrollers also exhibited decreased mRNA levels of *SLC16A3* (encoding Monocarboxylate transporter 4 [MCT4]), known to act as a H^+^-linked lactate transporter enabling lactate efflux in high glycolytic cells (20). Next to the downregulation of glycolysis-associated genes, we observed decreased expression levels of genes related to components of oxidative phosphorylation (OXPHOS), including *NDUFA4*, *COX6A1*, *COX14*, and *SDHB*. These genes encode components of complex I–III of the mitochondrial electron transport chain (ETC). Moreover, genes encoding for components of ATP synthase (complex V and *ATP5*), and expression of *PPARGC1*α encoding the transcriptional coactivator PGC1-α, a key regulator of mitochondrial biogenesis in T cells, were downregulated in noncontrollers (Figure 2, B–D, and G). Expression of genes involved in cellular defense against reactive oxygen species (ROS) (i.e., *NFE2L2*, *SOD2*,and *PRDX1*), as well as genes involved in lysosomal degradation (*CTSA* and *ATP6V1F*) were also downregulated in CMV-specific CD8^+^ T cells of noncontrollers (Figure 2, B–D, and Supplemental Figure 2B). In contrast, the rate-limiting enzyme of mitochondrial fatty acid oxidation (FAO), *CPT1a*, was increased in noncontrollers (Figure 2, B–D). To account for the fact that many of these genes are intertwined, we conducted pathway analysis and identified additional genes that were downregulated and related to components of glycolysis (e.g., *ALDOA*) and mitochondrial respiration (*NDUFA/B* homologs), or were upregulation and related to FAO (e.g., *ACADL*) in noncontrollers (Figure 2, E–G, and Supplemental Figure 2C).

### CMV-specific CD8^+^ T cells of noncontrollers exhibit restrained glycolytic capacity and mitochondrial respiration while fatty acid metabolism is increased.

**Figure 3:**
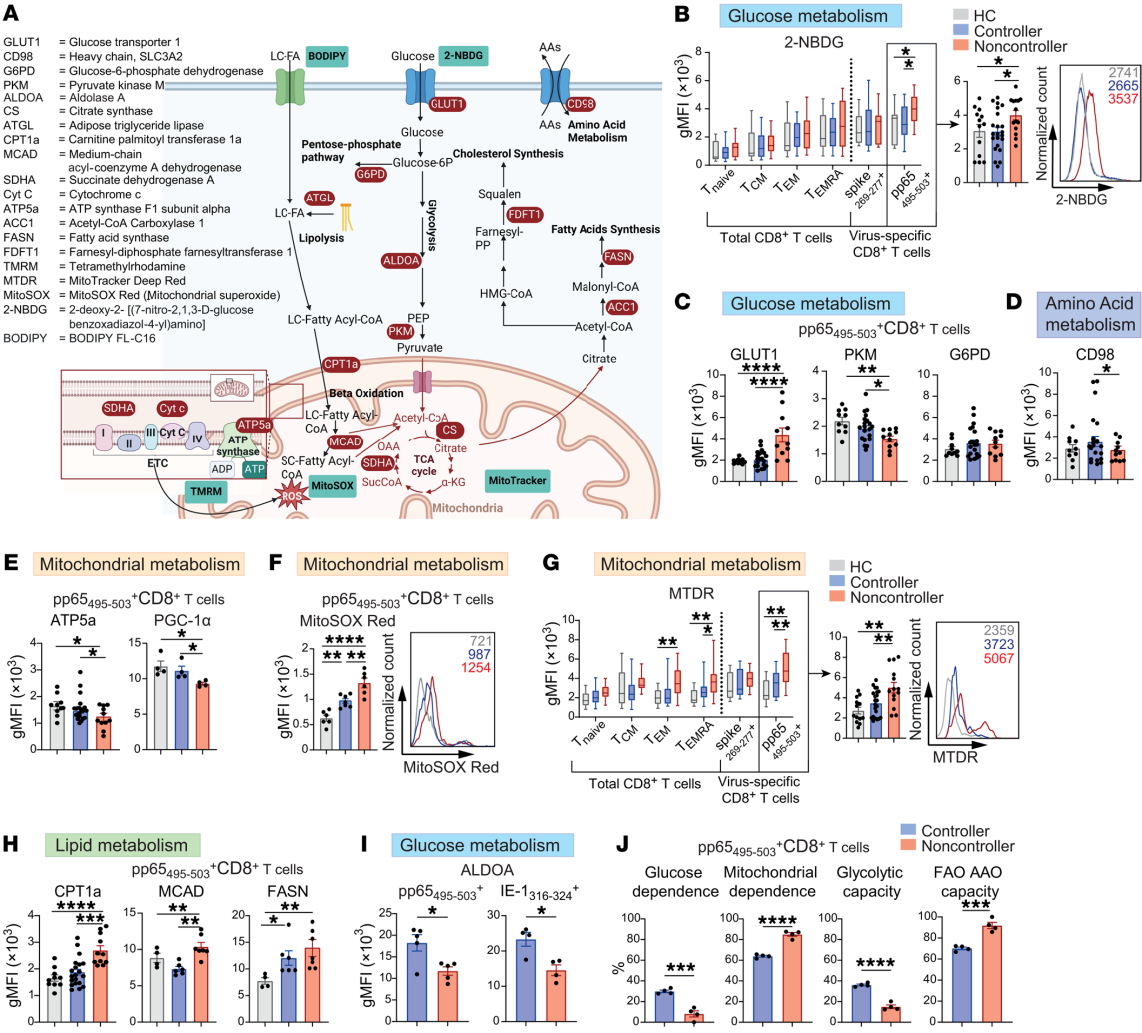
CMV-specific CD8^+^ T cells of noncontrollers exhibit restrained glycolytic capacity and mitochondrial respiration while fatty acid metabolism is increased. (**A**) Overview of metabolic targets (red) and metabolic probes (blue) for analysis by spectral flow cytometry. Created with biorender.com. (**B**–**I**) Ex vivo analysis of metabolic characteristics of CD8^+^ T cell subsets. (**B**) Geometric mean fluorescence intensity (gMFI) of 2-NBDG (13–22/group). (**C**) GLUT1, PKM, and G6PD expression of pp65_495–503_–specific CD8^+^ T cells (*n* = 10–20/group). (**D**) CD98 expression of pp65_495–503_–specific CD8^+^ T cells (*n* = 10–20/group). (**E**) ATP5a (*n* = 10–20/group) and PGC1α (*n* = 4/group) expression of pp65_495–503_–specific CD8^+^ T cells. (**F**) MitoSOX gMFI (n=6/group) of pp65_495–503_–specific CD8^+^ T cells. (**G**) MTDR gMFI (*n* = 13–22/group). (**H**) CPT1a (*n* = 10–20/group), MCAD, and FASN (*n* = 4–7/group) expression of pp65_495–503_–specific CD8^+^ T cells (**I**) Expression of ALDOA in pp65_495–503_ and IE-1_316–324_–specific CD8^+^ T cells within the same individuals (IE-1_316–324_–specific cells could not be detected in 1 individual) (*n* = 5/group). (**J**) SCENITH of pp65_495–503_–specific CD8^+^ T cells (*n* = 4/group). Data are presented as mean ± SEM or as boxplot (bounds of the boxes indicate upper and lower quartile, line indicates median, whiskers indicate min and max). Each dot represents an individual. Statistical analysis by 1-way ANOVA with Tukey’s test for multiple comparisons, 2-way ANOVA with Šidák’s test for multiple comparisons or 2-sided student’s *t* test. *P <0.05; **P < 0.01; ***P < 0.001; ****P < 0.0001.

To validate our transcriptional findings, we analyzed the metabolic states of virus-specific CD8^+^ T cell populations ex vivo at the protein level in single cells by using high-dimensional spectral flow cytometry (21). The interrogated metabolic components included enzymes, nutrient transporters, functional probes, and assays to interrogate core metabolic pathways, including glycolysis, pentose-phosphate pathway, amino acid uptake, mitochondrial respiration, TCA cycle, and fatty acid synthesis (Figure 3A).

For initial analysis of glucose-related metabolism we cultured virus-specific T cells ex vivo in the presence of the fluorescent glucose-analogue 2-NBDG, which serves as a surrogate for glucose uptake (22). In our study cohort, we detected an increased uptake of 2-NBDG by CMV-specific CD8^+^ T cells from noncontrollers compared with cells from HCs or KTRs controlling CMV (Figure 3B). No differences in 2-NBDG uptake were observed for SARS-CoV-2 spike_269–277_–specific CD8^+^ T cell populations, neither for naive nor for canonical memory CD8^+^ T cell subsets between HCs, controllers, and noncontrollers (Figure 3B and Supplemental Figure 3A). In line with the transcriptional data and increased 2-NBDG uptake, we detected a distinct upregulation of GLUT1 in CMV-specific CD8^+^ T cells of noncontrollers (Figure 3C). Corroborating our transcriptional data, CMV-specific CD8^+^ T cells of CMV noncontrollers displayed a decreased protein expression of PKM (Figure 3C). However, expression levels of glucose-6-phosphate dehydrogenase (G6PD), a crucial component of the pentose-phosphate pathway, which also requires glucose-6-phosphate, did not differ between CMV-specific CD8^+^ T cells of different patient groups (Figure 3C). In contrast to CMV-specific CD8^+^ T cells, SARS-CoV-2–specific CD8^+^ T cells did not differ in GLUT1 or PKM levels when comparing KTR CMV controllers with noncontrollers or HCs (Supplemental Figure 3B). Together, these findings indicate a distinct discordance between increased glucose uptake yet impaired downstream glycolytic enzyme expression in CMV-specific CD8^+^ T cells of CMV noncontrollers.

With respect to amino acid uptake, expression of the heterodimeric neutral amino acid transporter CD98 on CMV-specific CD8^+^ T cells was decreased in noncontrollers compared with controllers (Figure 3D), which is in accordance with decreased gene expression of *SLC7A5*, encoding for 1 part of the heterodimer, in noncontrollers (Figure 2, B–D). Also, in line with the transcriptomic analysis, the protein levels of ATP5a, a component of ATP synthase (ETC complex V, mitochondrial F1 complex), and PGC-1α were decreased in noncontrollers (Figure 3E). Expression of other mitochondrial proteins such as cytochrome c, CS, and SDHA, indicators for the electron transport chain and TCA cycle, was comparable in pp65_495–503_–specific CD8^+^ T cells of HCs, controllers, and noncontrollers, and these proteins were also not different in spike_269–277_–specific CD8^+^ T cells (Figure 3C and Supplemental Figure 3B. This discordance and the relationship of ATP synthase and mitochondrial biogenesis with mitochondrial stress (23) prompted us to directly interrogate if pp65_495–503_-specific CD8^+^ T cells of noncontrollers display signs of mitochondrial stress. Therefore, we stained these cells with MitoSOX Red, which measures mitochondrial-derived superoxide and can indicate mitochondrial stress (18). Superoxide levels were higher in noncontrollers compared with controllers and HCs (Figure 3F). Superoxide levels of spike_269–277_–specific CD8^+^ T cells, however, did not differ between the study groups (Supplemental Figure 3D). To further investigate mitochondrial characteristics, we measured the mitochondrial mass using MitoTracker Deep Red (MTDR) and the mitochondrial membrane potential using tetramethylrhodamine methyl ester (TMRM), both of which positively correlate with ROS levels/oxidative stress (24). Analysis of our cohort indeed showed increased MTDR and TMRM in CMV-specific CD8^+^ T cells from noncontrollers (Figure 3G and Supplemental Figure 3E). In contrast, spike_269–277_–specific CD8^+^ T cells revealed no differences between HCs, controllers, and noncontrollers, indicating that the mitochondrial alterations are a distinctive feature of CMV-specific CD8^+^ T cells (Figure 3, E and G, and Supplemental Figure 3E). Notably, MTDR and TMRM were also enhanced in the total CD8^+^ T_EM_ and T_EMRA_ populations (Figure 3G and Supplemental Figure 3E), which may be caused by inclusion of numerous other CMV-specific T cells in these 2 populations (9). Consistent with the transcriptional data, the expression of FAO components CPT1a and MCAD were increased in CMV-specific T cells of noncontrollers (Figure 3H). Further, the fatty acid synthesis enzymes ACC1 and FASN were increased in CMV-specific CD8^+^ T cells of noncontrollers (Figure 3H and Supplemental Figure 3C). In contrast, CPT1a and ACC1 were not altered in spike_269–277_–specific CD8^+^ T cells, underscoring an exclusive effect by uncontrolled CMV infection (Supplemental Figure 3B). Differences in the levels of ATGL, which catalyzes the intracellular release of fatty acids from triacylglycerol and FDFT1, a cholesterol biosynthesis enzyme, were not detected in CMV-specific CD8^+^ T cells (Supplemental Figure 3C). Uptake of fatty acids, measured by uptake of BODIPY-FL-C16, was also not altered (Supplemental Figure 3F). Analysis of metabolic markers in noncontrollers based on intermediate or high CMV infection risk showed an increased GLUT1 expression in CMV-specific CD8^+^ T cells of patients who are high risk for CMV, whereas no differences were detected in PKM, ATP5a, MTDR, and CPT1a (Supplemental Figure 3G).

To determine whether the metabolic alterations observed in pp65_495–503_–specific CD8^+^ T cells are also present in CD8^+^ T cells specific for another CMV epitope, we analyzed IE-1_316–324_–specific CD8^+^ T cells and detected a comparable decrease in the expression of aldolase (ALDOA) and ATP5a in both virus-specific CD8^+^ T cell populations of noncontrollers compared with controllers (Figure 3I and Supplemental Figure 3H). To characterize differences in metabolic capacities and dependencies we performed SCENITH, a flow cytometry–based method to functionally profile energy metabolism at single-cell resolution (25). At baseline, we observed a lower puromycin incorporation in pp65_495–503_–specifc CD8^+^ T cells in noncontrollers than in controllers underscoring the reduced expression of ATP5a as part of ETC complex V, which is the main ATP generator in resting memory CD8^+^ T cells (26) (Supplemental Figure 3I). In accordance with the decreased expression of PKM and ALDOA, a lower glucose dependence and glycolytic capacity in pp65_495–503_–specifc CD8^+^ T cells of noncontrollers was detected (Figure 3J). In contrast, an increased dependence on mitochondrial ATP and an increased capacity of FAO and amino acid oxidation (AAO) were recorded in noncontrollers (Figure 3J), which is in line with the increased expression of CPT1a and MCAD. Taken together, these data indicate that CMV-specific CD8^+^ T cells in noncontrollers exhibit increased FAO/fatty acid synthesis while, concurrently, glycolytic capacity and mitochondrial functionality were perturbed at both the transcriptional and protein level.

### Impaired glycolytic responsiveness but increased FAO dependency of antigen-stimulated CMV-specific CD8^+^ T cells in noncontrollers.

**Figure 4:**
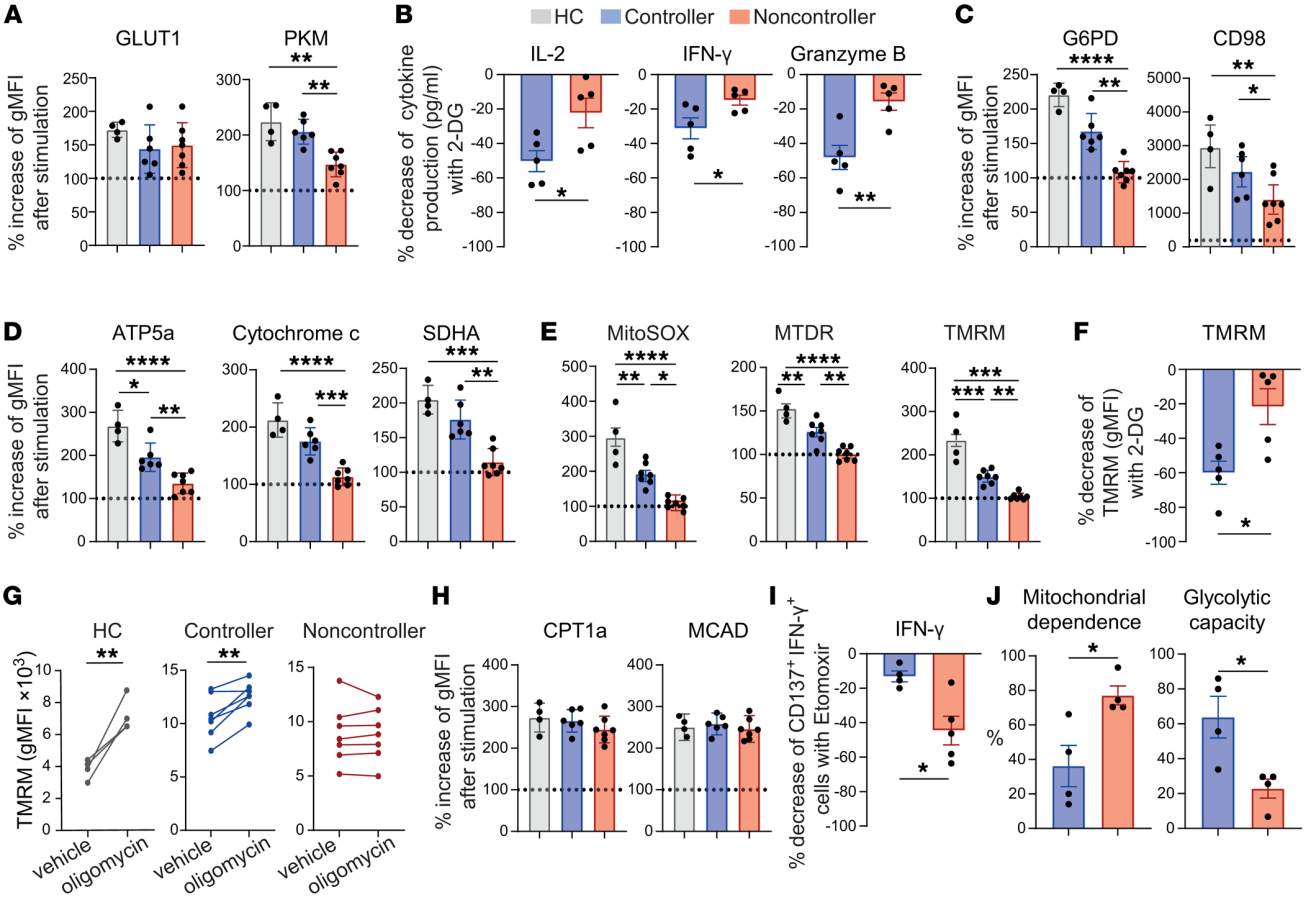
Impaired glycolytic responsiveness and increased FAO dependency of antigen-stimulated CMV-specific CD8^+^ T cells in noncontrollers. (**A**–**I**) PBMCs were stimulated with pp65_495–503_ peptide for either 20 hours (short stimulation) or 6 days (long stimulation). (**A**) Relative change in gMFI to unstimulated condition of GLUT1 and PKM expression of pp65_495–503_–specific CD8^+^ T cells after long stimulation (*n* = 4–7/group). (**B**) After short stimulation, supernatant was collected for the analysis of cytokine and granzyme production. Cells were either cultured in presence of 2-DG or not. The percentage decrease in cytokine concentration (pg/mL) after 2-DG treatment is shown (*n* = 5/group). (**C** and **D**) Relative change (to unstimulated condition) of G6PD and CD98 expression (**C**), ATP5a, cytochrome c, and SDHA expression (**D**) of pp65_495–503_–specific CD8^+^ T cells after long stimulation (*n* = 4–7/group). (**E**) Relative change (to unstimulated condition) of MitoSOX, MTDR, and TMRM of pp65_495–503_–specific CD8^+^ T cells after short stimulation (*n* = 4–7/group). (**F**) Cells were long stimulated and cultured either in the presence or absence of 2-DG. The percentage decrease of TMRM in CD137^+^CD8^+^ T cells by 2-DG treatment is shown. (**G**) Cells were long stimulated in presence or absence of oligomycin. Relative change (stimulated/stimulated + oligomycin) of TMRM uptake in CD137^+^CD8^+^ T cells (*n* = 4–7/group). (**H**) Relative change (to unstimulated condition) of CPT1a and MCAD expression of pp65_495–503_–specific CD8^+^ T cells after long stimulation (*n* = 4–7/group). (**I**) After short stimulation, cytokine production was measured by intracellular cytokine staining. Percentage decrease of CD137^+^IFN-γ^+^ cells (of total CD8^+^ T cells) in the presence of etomoxir (*n* = 4–5/group). (**J**) SCENITH of CD137^+^CD69^+^CD8^+^ T cells after 4 hours of stimulation with pp65_495–503_ peptide (*n* = 4/group). Data are presented as mean ± SEM. Each symbol represents an individual. Statistical analysis by 2-sided student’s *t* test or 1-way ANOVA with Tukey’s test for multiple comparisons. *P < 0.05; **P < 0.01; ***P < 0.001; ****P < 0.0001.

Corresponding with the impaired ability of noncontrollers to produce cytokines, CMV-reactive CD137^+^CD8^+^ T cells of noncontrollers displayed a substantial inability to upregulate PKM after antigenic stimulation compared with HCs and controllers, whereas GLUT1 expression responded comparably to stimulation (Figure 4A). On the other hand, spike_269–277_–reactive CD8^+^ T cells of controllers and noncontrollers displayed a similar increase in the expression of PKM (Supplemental Figure 4A).

Given the impaired responsiveness of PKM in CMV-specific CD8^+^ T cells of noncontrollers after stimulation, we assessed the impact of blocking glycolysis with 2-Deoxyglucose (2-DG). The decrease in production of IL-2, IFN-γ, and Granzyme B by CMV-specific CD8^+^ T cells was apparent in controllers, but noncontrollers were less affected by glycolysis blockade (Figure 4B). TNF and perforin production was less sensitive for glycolysis blockade and was similarly affected in controllers and noncontrollers (Supplemental Figure 4B). In addition, HCs and KTRs controlling CMV infection responded with a stronger upregulation of G6PD and CD98 than noncontrollers (Figure 4C).

The inferior increase after antigenic stimulation for proteins related to glycolysis, pentose-phosphate pathway, and amino acid uptake in noncontrollers was accompanied by impaired increases in the expression of ETC components, superoxide production, and the mitochondrial mass and polarization when compared to HCs and controllers (Figure 4, D and E, and Supplemental Figure 4C). Glycolysis inhibition by 2-DG resulted also in a less pronounced decrease of TMRM in noncontrollers (Figure 4F). To further assess the mitochondrial capacity, we coincubated cells with the ATP synthase inhibitor oligomycin. In healthy mitochondria, blockade of ATP production leads to an increase of membrane potential due to inhibition of proton reentry via ATP synthase, while dysfunctional mitochondria react with stable or even declined membrane potential (27). CMV-reactive CD8^+^ T cells from HCs and controllers reacted with an increased TMRM, whereas no increase could be provoked in noncontrollers (Figure 4G). Thus, both the cytokine production and mitochondrial membrane potential of CMV-specific CD8^+^ T cells in noncontrollers are less affected by inhibition of glycolysis and ATP production.

The increase in expression of CPT1a, MCAD, ACC1, FASN, and FDFT1 after stimulation of CMV-specific CD8^+^ T cells was similar between the study groups (Figure 4H and Supplemental Figure 4D). Given the yet comparable relative increase of these components after stimulation in noncontrollers, regardless of the initial higher levels, we questioned whether CMV-specific CD8^+^ T cells from noncontrollers would instead rely more on FAO. To this end, we stimulated these cells in presence of CPT1a inhibitor etomoxir and determined the ability to produce IFN-γ. After inhibition of CPT1a, the IFN-γ production of CD8^+^ T cells was more diminished in noncontrollers (Figure 4I). To further validate the observed differences in the stimulation-induced upregulation of metabolic protein expression and the dependence on glycolysis or FAO between controllers and noncontrollers, we performed SCENITH after PBMCs were stimulated for 4 hours by pp65_495–503_ peptide (Figure 4J and Supplemental Figure 4E). We observed a greater mitochondrial dependence by the stimulated CMV-specific CD8^+^ T cells of noncontrollers compared with controllers, which was accompanied by a decreased glycolytic capacity. In conclusion, noncontrollers appear to rely more on FAO, whereas controllers are more dependent on glycolysis.

### CD38 expression is particularly elevated on active circulating CMV-specific CD8^+^ T cells and is associated with metabolic dysfunction.

**Figure 5:**
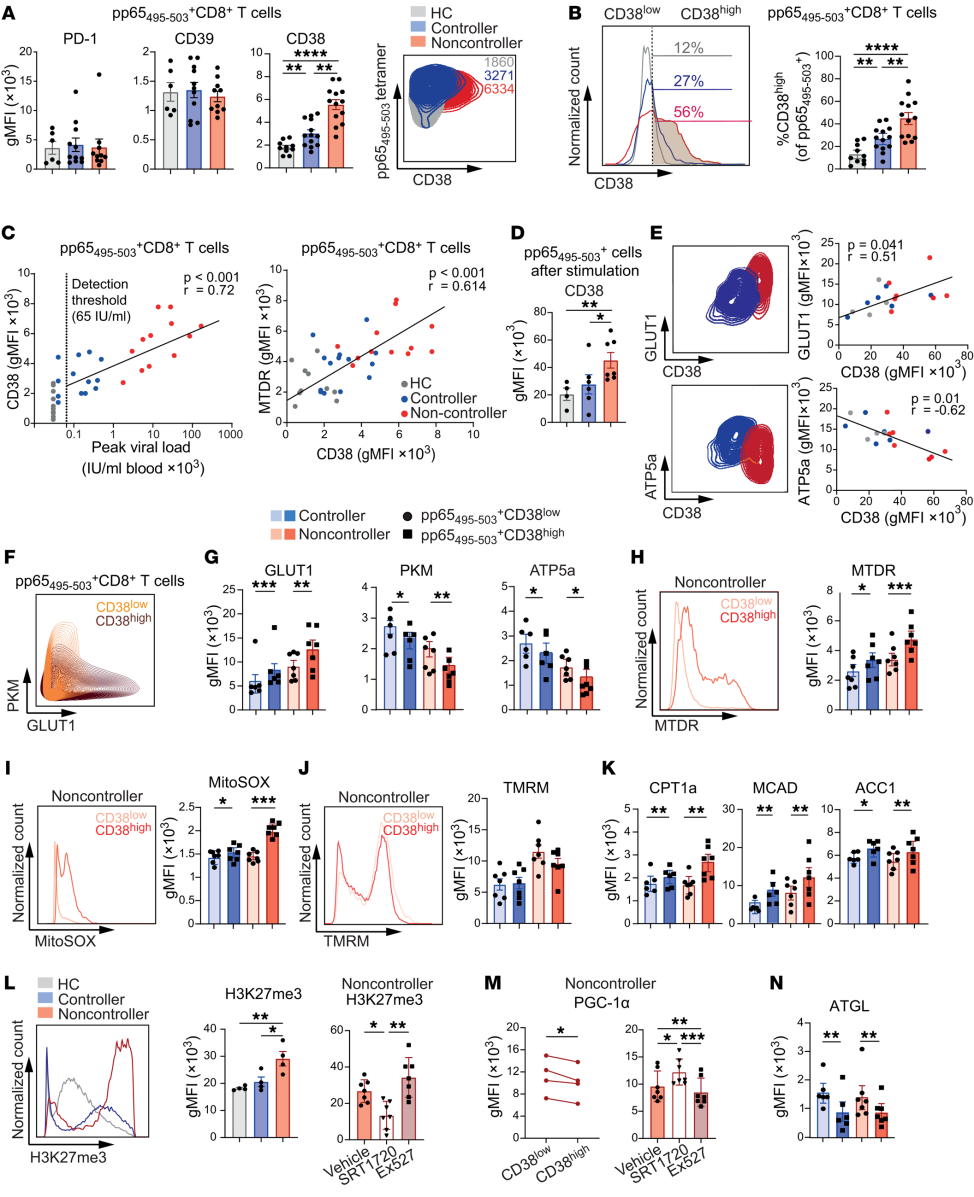
CD38 expression is particularly elevated on active circulating CMV-specific CD8^+^ T cells and associates with metabolic dysfunction. (**A**) Expression of PD-1, CD39 (*n* = 6–12/group), and CD38 (*n* = 10–13/group) on pp65_495–503_–specific CD8^+^ T cells. (**B**) Percentage of CD38^hi^ pp65_495–503_–specific CD8^+^ T cells (*n* = 10–13/group). (**C**) Correlation between CD38 expression of pp65_495–503_–specific CD8^+^ T cells with the highest detected CMV load in blood and MTDR uptake (*n* = 10–13/group). (**D** and **E**) PBMCs were stimulated for 6 days with pp65_495–503_ peptide. (**D**) CD38 expression of pp65_495–503_–specific CD8^+^ T cells. (**E**) Expression of GLUT1 versus CD38 and ATP5a versus CD38 (*n* = 4–7/group). (**F**) Expression of PKM versus GLUT1. (**G**) GLUT1, PKM, and ATP5a expression of CD38^lo^ and CD38^hi^ pp65_495–503_–specific CD8^+^ T cells. (**H**–**J**) MTDR (**H**), MitoSOX (**I**), and TMRM (**J**) staining of CD38^lo^ and CD38^hi^ pp65_495–503_–specific CD8^+^ T cells. (**K**) CPT1a, MCAD, and ACC1 expression of CD38^lo^ and CD38^hi^ pp65_495–503_–specific CD8^+^ T cells. (**L**) Histogram and left bar graph show H3K27me3 levels in pp65_495–503_–specific CD8^+^ T cells (*n* = 4/group). Right bar graph shows H3K27me3 levels in pp65_495–503_–specific CD8^+^ T cells of noncontrollers (*n* = 7) after treatment with NMN, SRT1720, or Ex527. (**M**) Left: PGC-1α expression in CD38^lo^ and CD38^hi^ pp65_495–503_–specific CD8^+^ T cells (*n* = 4). Right: PGC-1α levels in pp65_495–503_–specific CD8^+^ T cells of noncontrollers after treatment with NMN, SRT1720, or Ex527 (*n* = 7). (**N**) ATGL expression of CD38^lo^ and CD38^hi^ pp65_495–503_–specific CD8^+^ T cells. Data are presented as mean ± SEM. Each dot represents an individual. Statistical analysis by 1-way ANOVA with Tukey’s test for multiple comparisons, repeated-measures ANOVA with Geisser-Greenhouse correction, paired, 2-tailed *t* test, or Pearson correlation. *P < 0.05; **P < 0.01; ***P < 0.001; ****P < 0.0001.

Given the activated T cell state, based on the transcriptomic data (i.e., elevated expression of *GZMB*, *NKG7*, and *ZAP70*) and the observed distinctive metabolic alterations of CMV-specific CD8^+^ T cells from noncontrollers, we investigated the expression levels of the following cell surface markers that relate to (chronic) T cell activation and are known to effect cellular metabolism: PD-1, known for its effect on T cell metabolism (e.g., through repression of glycolysis (28)), CD39, an ectoenzyme catalyzing the hydrolyzation of adenosine tri- and diphosphate to adenosine monophosphate (29), and CD38, an ectoenzyme with NADase and ADP-ribosyl cyclase activity, thereby controlling NAD^+^ levels (30). No differences in the expression of both PD-1 and CD39 on pp65_495–503_–specific CD8^+^ T cells were observed between the study groups (Figure 5A). In contrast, upregulation of CD38 expression was evident on pp65_495–503_–specific CD8^+^ T cells of noncontrollers (Figure 5A), which was even more pronounced in individuals who were at high risk for CMV (Supplemental Figure 5A). Moreover, the fraction of CD38^hi^ cells within the population of pp65_495–503_–specific CD8^+^ T cells was increased in noncontrollers compared with controllers and HCs (Figure 5B). Notably, the percentages of CD38^hi^ pp65_495–503_–specific CD8^+^ T cells of controllers were also increased compared with HCs (Figure 5B). CD38 expression was also elevated on IE-1_316–324_–specific CD8^+^ T cells (Supplemental Figure 5B), whereas its expression on spike_269–277_–specific CD8^+^ T cells was equivalent between all groups (Supplemental Figure 5C).

To gain deeper insight into the instructions of the viral context in driving CD38 expression on virus-specific CD8^+^ T cells, we analyzed CD38 expression data from our previously published study in which we investigated responses at day 50 after infection in controlled experimental models of acute lymphocytic choriomeningitis virus–Armstrong strain (LCMV-Armstrong), low-level persistent (mouse CMV [MCMV]) and high-level chronic (LCMV-clone 13) viral infection using mass cytometry (31). The employed MCMV strain expressed the GP_33–41_ epitope from LCMV under the control of the ie2 promoter, allowing to study inflationary CD8^+^ T cells and compare these with responses to the same epitope present in acute and chronic LCMV. Persistent CMV infection drove high expression of CD38 on antigen-specific CD8^+^ T cells. CD38 expression on CMV-specific CD8^+^ T cells was enriched in secondary lymphoid tissues, i.e., spleen and bone marrow, and even reached higher levels in the spleen compared with the viral-specific T cells elicited after chronic LCMV-clone 13 infection (Supplemental Figure 5D). In the liver, where CD38 expression marks the tissue-resident viral-specific CD8^+^ T cells, a lesser distinction between the different infection types was observed (Supplemental Figure 5D). PD-1 was predominantly expressed on viral-specific CD8^+^ T cells during LCMV-clone 13 infection in all tissues, while this molecule was largely absent after LCMV-Armstrong and MCMV infection (Supplemental Figure 5E). Compared with CD38, CD39 exhibited lower expression on viral-specific CD8^+^ T cells after MCMV infection. The frequency of CD39^+^ virus-specific T cells after LCMV-clone 13 infection, however, was distinctly elevated in the spleen and liver (Supplemental Figure 5E). To determine whether viral replication drives the elevated CD38 expression on circulating CMV-specific T cells, we analyzed viral-specific T cells after infection with either a WT strain or a single-cycle replicating MCMV strain (MCMV-FKBP), both expressing the same model antigen E7 from human papilloma virus. The persistently replicating WT strain provoked higher CD38 expression on the circulating E7_49–57_–specific CD8^+^ T cells in the spleen, which indicates that enduring antigenic triggering elevated CD38 expression (Supplemental Figure 5F). Together, these data underscore that active CMV infection in mice and humans lead to a particular increase of CD38 expression by virus-reactive CD8^+^ T cells.

As CD38 constitutes a distinctive cell-surface marker with a potential metabolic impact, we next analyzed the clinical data on CMV replication in the blood of KTRs and assessed correlations with CD38 expression and metabolic function of CMV-specific CD8^+^ T cells. We observed a positive correlation between CD38 expression levels and peak viral load quantities in the circulation (Figure 5C). Further, we observed a strong correlation between mitochondrial mass and CD38 expression (Figure 5C) and a moderate correlation between TMRM and CD38 expression (Supplemental Figure 5G).

Next, we investigated the effects of antigenic stimulation on CD38 expression and the corresponding metabolic adaptations. As expected, CD38 expression on CMV-specific CD8^+^ T cells increased after stimulation. Remarkably, CD38 expression remained elevated on these T cells in noncontrollers (Figure 5D), which may indicate an altered regulation of CD38 expression leading to overexpression. The increased CD38 expression after stimulation positively correlated with GLUT1 but inversely correlated with PKM and ATP5a (Figure 5E and Supplemental Figure 5H). No correlation between CD38 expression and CPT1a was observed (Supplemental Figure 5H).

Given the elevated CD38 expression in noncontrollers, we investigated whether the metabolic alterations were also present when comparing CD38^lo^ and CD38^hi^ pp65_495–503_–specifc CD8^+^ T cells within KTR controllers and noncontrollers ex vivo. We detected GLUT1 upregulation of the CMV-specific CD38^hi^ compared with CD38^lo^ CD8^+^ T cells in controllers and noncontrollers (Figure 5, F and G). In contrast, we observed a decreased expression of CD98 in CD38^hi^CD8^+^ T cells (Supplemental Figure 5I). Moreover, we also observed lower PKM and ATP5a levels in the CMV-specific CD38^hi^CD8^+^ T cells compared with their CD38^lo^ counterparts, whereas the expression of G6PD revealed no differences (Figure 5, F and G, and Supplemental Figure 5I). Correspondingly, increased mitochondrial mass and superoxide levels were detected in the CD38^hi^ pp65_495–503_–specific CD8^+^ T cells, which was particularly prominent in noncontrollers (Figure 5, H and I). No differences in TMRM were observed between CD38^hi^ and CD38^lo^ cells (Figure 5J). Analysis of the mitochondrial proteins cytochrome c and CS did not reveal differences between CD38^hi^ and CD38^lo^ cells (Supplemental Figure 5I). Investigation of lipid metabolism revealed an increased expression of CPT1a, MCAD, and ACC1 in CD38^hi^ CMV-specific CD8^+^ T cells (Figure 5K).

The level of H3K27me3 serves as an indirect marker of CD38/NAD/SIRT1/EZH2 axis activity, in which increased CD38 expression leads to NAD^+^ reduction, causing SIRT1 inhibition followed by increased EZH2 acetylation and trimethylation of Lys-27 in histone 3 (H3K27me3) (30). To determine whether altered CD38 expression was functionally relevant for CMV-specific CD8^+^ T cells, we determined its downstream influence on H3K27me3. Higher levels of H3K27me3 were most profoundly detected in CMV-specific CD8^+^ T cells of non-controllers. Moreover, treatment with the SIRT1 agonist SRT1720 reduced H3K27me3 in noncontrollers (Figure 5L). Conversely, addition of the SIRT1 antagonist Ex527 increased H3K27me3, underlining the impact of NAD^+^ availability and SIRT1 function on H3K27me3 status (Figure 5L). In addition, we recorded a lower expression of PGC-1α in CD38^hi^pp65_495–503_^+^ cells of noncontrollers, which increased after treatment with SRT1720 and decreased after addition of Ex527 (Figure 5M). As NAD^+^ enhances ATGL expression through increased SIRT1 activity (32, 33), we additionally assessed ATGL expression levels. Indeed, ATGL expression was lower in CD38^hi^ CMV-specific CD8^+^ T cells in both controllers and noncontrollers (Figure 5N).

Next, we asked if CD38 expression was also increased on virus-specific CD8^+^ T cells upon acute infection at an early time point. Therefore, we investigated individuals from a cohort study (no transplant patients), who experienced SARS-CoV-2 infection. As these individuals were already vaccinated against SARS-CoV-2, we could compare spike-specific CD8^+^ T cells before and during acute infection (within 7 days after symptom onset). We recorded both an increased CD38 expression and a fraction of CD38^hi^ cells within the spike-specific CD8^+^ T cell population during acute infection (Supplemental Figure 5J). Further, an increased expression of GLUT1 in spike-specific CD8^+^ T cells during acute infection was observed, but in contrast to CMV-specific T cells this was accompanied by increases in aldolase and ATP5a expression (Supplemental Figure 5K). We also observed increases in GLUT1, aldolase, and ATP5a expression in CD38^hi^ compared with CD38^lo^ spike-specific CD8^+^ T cells in individuals during acute infection (Supplemental Figure 5L).

All together, we conclude that CD38 expression is particularly elevated in the setting of CMV infection. Moreover, this molecule associates with metabolic dysfunction and a distinct, yet reversible, epigenetic state on CMV-specific CD8^+^ T cells. In contrast, during acute infection with SARS-CoV-2, an upregulation of CD38 on virus-specific CD8^+^ T cells was associated with an activated metabolic state, indicating a differential CD8^+^ T cell metabolism during acute versus chronic infection.

### Elevated expression of CD38 on CMV-specific CD8^+^ T cells during viral persistence gradually rewires cellular metabolism.

**Figure 6:**
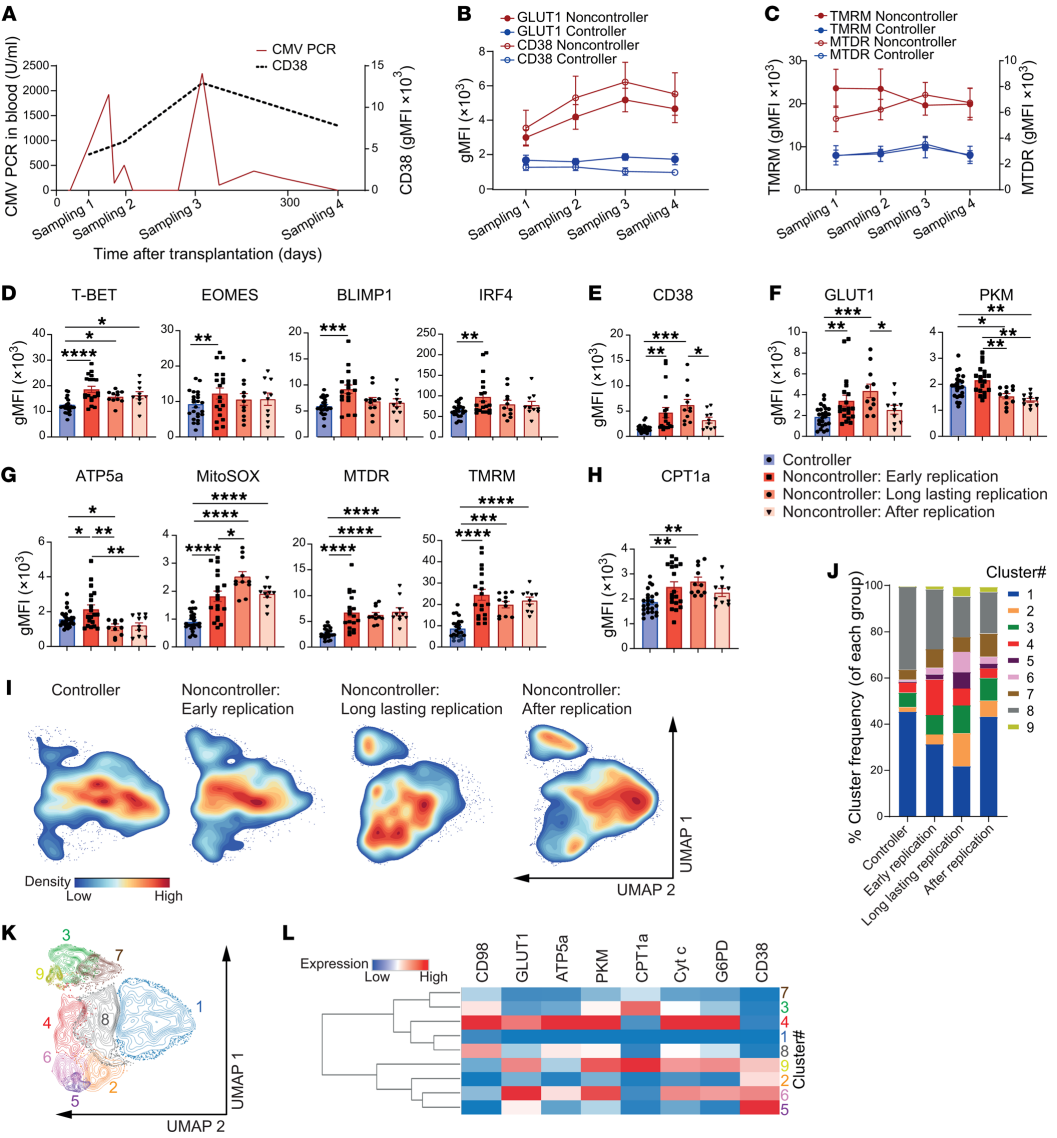
Elevated expression of CD38 on CMV-specific CD8^+^ T cells during viral persistence gradually rewires cellular metabolism. An independent cohort of 16 patients was longitudinally analyzed after kidney transplantation. Blood samples for analysis of pp65_495–503_–specific CD8^+^ T cells were taken at 6 weeks (sampling 1), 3 months (sampling 2), 6 months (sampling 3), and 12 months (sampling 4) after transplantation. CMV replication in blood was continuously assessed for 18 months. (**A**) Representative graph shows the CMV load in blood (left, y axis) and the expression of CD38 (right, y axis) over time. (**B** and **C**) Longitudinal expression of GLUT1 and CD38 (**B**) and TMRM and MTDR levels (**C**) of pp65_495–503_–specific CD8^+^ T cells from controllers (blue, *n* = 6) and noncontrollers (red, *n* = 10). (**D**–**G**) Expression of the transcription factors T-BET, EOMES, BLIMP-1, and IRF-4 (**D**), CD38 (**E**), GLUT1 and PKM (**F**), ATP5a, MitoSOX, MTDR, and TMRM (**G**), and CPT1a (**H**) of pp65_495–503_–specific CD8^+^ T cells from controllers and noncontrollers (subdivided according to onset of viral replication detection: early, long lasting, and after). (**I**–**L**) High-dimensional phenotypical analysis pp65_495–503_–specific CD8^+^ T cells from controllers and noncontrollers (early, long lasting, and after viral replication). (**I**) UMAP plots showing the density of metabolic protein and CD38 expression. (**J**) Stacked bar graph of 9 FlowSOM clusters per group. (**K**) FlowSOM consensus meta-clustering with 9 clusters. (**L**) Hierarchically clustered heatmap of the metabolic phenotypes of the clusters shown in **J** and **K**. Marker expression is shown per cluster as z score of median signal intensity per channel. Data are presented as mean ± SEM. Each symbol in **D**–**H** represents an individual. Statistical analysis by 1-way ANOVA with Tukey’s test for multiple comparisons. *P < 0.05; **P < 0.01; ***P < 0.001; ****P < 0.0001.

To obtain better insight into the interplay between the stage of CMV infection and the metabolic characteristics of CMV-specific CD8^+^ T cells, we analyzed blood samples of an independent KTR cohort longitudinally. We divided the cohort based on the aforementioned criteria into controllers (*n* = 6) and noncontrollers (*n* = 10). Like in the main cohort, the blood values of tacrolimus and MPA did not differ between both longitudinally assessed groups (Supplemental Figure 6A). Visualization of CD38 expression and viremia in noncontrollers revealed fluctuating values in individual patients over time, yet, on average CD38, GLUT1, TMRM, and mitochondrial mass increased upon (concurrent) CMV replication (Figure 6, A–C, and Supplemental Figure 6B). Notably, the peak of CD38 expression on CMV-specific CD8^+^ T cells corresponded with increased CMV viremia.

For comparative analysis between groups, all samples (sampling 1–4) from controllers, were combined as we observed very low variation in CD38 expression and metabolic markers (exemplary shown for GLUT1, TMRM, and MTDR) over time per individual (Figure 6, B and C). With respect to the onset of viral replication, we subclassified all samples (sampling 1–4) from noncontrollers in 3 categories: (a) early replication: samples taken between 1 week before until 2 weeks after replication onset; (b) long lasting replication: samples taken more than 2 weeks after replication onset with still detectable replication (until 2 weeks after last positive CMV-PCR); and (c) after replication: samples taken at least 2 weeks after last detectable replication. To validate the subclassification we analyzed the expression of the transcription factors T-BET, EOMES, BLIMP-1, and IRF4, which all connect to CMV infection (34) in the pp65_495–503_–specific CD8^+^ T cells during the different stages of replication (Figure 6D). At the early replication stage, we detected an increased expression of T-BET, EOMES, BLIMP-1, and IRF-4, compared with controllers, indicating enhanced effector/effector-memory formation in the early phase. Expression of T-BET slightly declined but remained elevated at later time-points after CMV replication, while expression of EOMES, BLIMP-1, and IRF4 expression declined to similar levels compared with controllers. Thus, there is indeed an association between the induction of CMV associated transcription factors and the onset of CMV replication, and the increased T-BET levels longitudinally indicate CMV replication, which is in alignment with previous reports (34, 35).

CD38 expression increased with ongoing CMV viremia followed by a drop after viral clearance (Figure 6E). We detected a similar pattern of expression for GLUT1, whereas PKM and ATP5a peaked at early replication but then declined in noncontrollers (Figure 6, F and G). Further, G6PD and cytochrome c levels remained relatively stable while CD98 expression gradually declined (Supplemental Figure 6C). The superoxide levels followed a similar pattern as CD38 and GLUT1 expression whereas the mitochondrial mass and membrane potential were elevated during early and late stages of CMV replication and beyond (Figure 6G). The increase of CTP1a expression also followed the same pattern as CD38/GLUT1/superoxide expression (Figure 6H).

To gain in-depth insight into the kinetics of CD38 expression and the different metabolic signatures, high-dimensional analysis of pp65_495–503_–specific CD8^+^ T cells by unsupervised UMAP and clustering was performed with the different stages of CMV replication (Figure 6, I–L, and Supplemental Figure 6D). At early replication, we observed a shift from CD38^lo^ metabolically less active clusters (no. 1 and no. 8), to an augmented frequency of clusters with increased CD38 expression and an intermediate (no. 2, no. 5) or high expression of proteins related to glycolysis, glucose uptake, and mitochondrial metabolism (no. 6, no. 9). In addition, another CD38^lo^ cluster (no. 4), marked by upregulated proteins related to glycolysis, glucose/amino acid uptake, and mitochondrial metabolism appeared. Longer lasting CMV replication was characterized by a further decline of less metabolically active clusters, whereas the prevalence of the CD38-expressing clusters increased (no. 2, no. 5, no. 6, no. 9), of which no. 2 and no. 5 were marked by low expression of metabolic proteins. Interestingly, this was accompanied by a contraction of the metabolic highly active cluster no. 4. After clearance of CMV replication, a reduction of CD38^hi^-expressing clusters occurred while the CPT1a^+^ cluster no. 3 remained present and the metabolically less active cluster no. 1 bounced back to the level of controllers. Together, these results indicate that (chronic) elevated expression of CD38 on CD8^+^ T cells in noncontrollers relates to a gradual rewiring of cellular metabolism characterized by a repressed expression of PKM and ATP5a but increased CPT1a expression compared with controllers.

### CD38 inhibition restores metabolic dysregulation and improves functionality of CMV-specific CD8^+^ T cells in noncontrollers.

Next, we asked whether the impaired cytokine functionality and metabolic alterations in CMV-specific CD8^+^ T cells from noncontrollers can be reversed. Given the signs of alterations in glycolytic capacity and mitochondrial stress, both of which are coupled to CD38 activity, we treated pp65_495–503_–specific CD8^+^ T cells ex vivo with either mitochondrial antioxidants, i.e., MitoQuinone (MQ) and MitoTempo (MT), or the CD38 inhibitor 78c (referred hereafter as CD38i), which is an uncompetitive inhibitor of CD38, inhibiting its hydrolase activity and leading to increased levels of NAD^+^ (36). However, treatment of stimulated CMV-specific CD8^+^ T cells with MQ or MT did not affect mitochondrial mass, mitochondrial membrane potential, and cytokine production in our study (Supplemental Figure 7, A and B).

**Figure 7:**
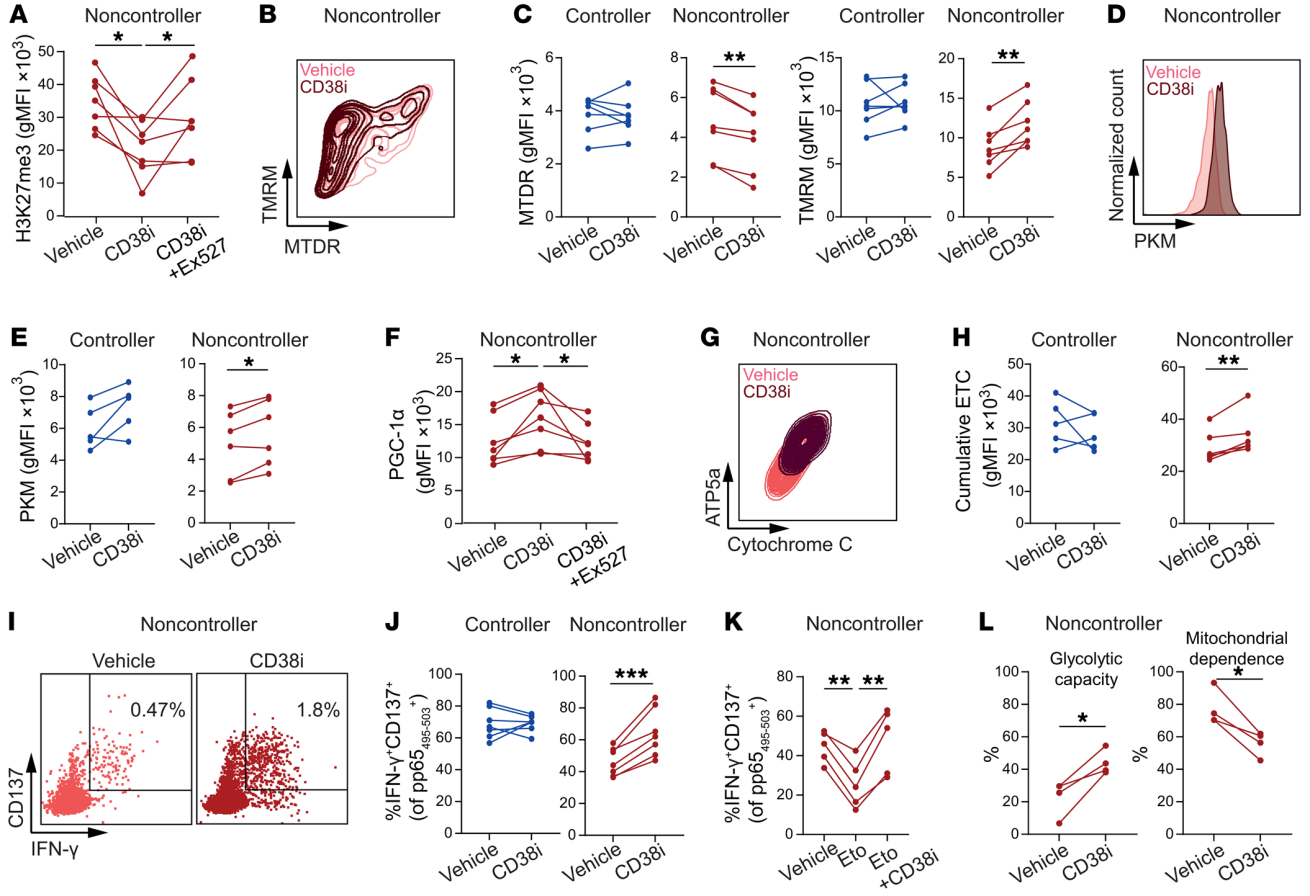
CD38 inhibition restores metabolic dysregulation and improves functionality of CMV-specific CD8^+^ T cells in noncontrollers. (**A**–**H**) PBMCs were stimulated with pp65_495–503_ peptide for either 20 hours (short stimulation) or 6 days (long stimulation). (**A**) H3K27me3 levels in long-stimulated pp65_495–503_–specific CD8^+^ T cells from noncontrollers (*n* = 7) in presence of vehicle, CD38i, or CD38i + Ex527. (**B** and **C**) Representative contour plot (**B**) and quantitative graphs (**C**) show MTDR and TMRM staining of pp65_495–503_–specific CD8^+^ T cells after short stimulation in the presence or absence of CD38i (*n* = 7/group). (**D** and **E**) Representative histogram (**D**) and quantitative graphs (**E**) show PKM expression of pp65_495–503_–specific CD8^+^ T cells after long stimulation in presence or absence of CD38i (*n* = 5–6/group). (**F**) Expression of PGC-1α in long-stimulated pp65_495–503_–specific CD8^+^ T cells from noncontrollers (*n* = 7) in presence of vehicle, CD38i, or CD38i + Ex527. (**G** and **H**) ATP5a and cytochrome c expression (**G**) and cumulative ETC protein expression (cytochrome c/SDHA/ATP5a) (**H**) after long stimulation in presence or absence of CD38i (*n* = 5–6/group). (**I** and **J**) After short stimulation, cytokine production was measured by intracellular cytokine staining in presence or absence of CD38i. Representative CD137 versus IFN-γ staining (**I**), percentage IFN-γ^+^CD137^+^ of pp65_495–503_–specific CD8^+^ T cells (*n* = 7/group) (**J**). (**K**) Percentage IFN-γ^+^CD137^+^ cells of pp65_495–503_–specific CD8^+^ T cells from noncontrollers after long stimulation with peptide in presence of vehicle, etomoxir, or etomoxir plus CD38i (*n* = 5). (**L**) PBMCs were cultured in presence of CD38i or vehicle for 16 hours, followed by stimulation with pp65_495–503_ peptide for 4 hours. Subsequently, SCENITH was performed on CD137^+^CD69^+^CD8^+^ T cells (*n* = 4 noncontrollers). Data are presented as mean ± SEM. Each symbol represents an individual. Statistical analysis by paired *t* test or repeated-measures ANOVA with Geisser-Greenhouse correction. *P < 0.05; **P < 0.01; ***P < 0.001.

To assess the impact of CD38 inhibition, we first analyzed the effect of CD38i on H3K27me3 levels of antigen-stimulated CMV-specific CD8^+^ T cells from noncontrollers. CD38i decreased H3K27me3 levels, indicating effective CD38 inhibition, which was reversed by a supplementary treatment with SIRT1 inhibitor Ex527 (Figure 7A). In contrast to the mitochondrial antioxidants, we observed a decrease of mitochondrial mass in the CMV-specific CD8^+^ T cells of noncontrollers (Figure 7, B and C), which may relate to restored autophagy of dysfunctional mitochondrial aggregates reported previously (37). The observed increase in mitochondrial membrane potential (Figure 7C) likely reflects a restored responsiveness upon stimulation compared with the impaired response that we observed without CD38i addition in noncontrollers (Figure 4, E and G). Further, we detected an increase of PKM in noncontrollers after CD38i treatment compared with nontreated stimulated cells (Figure 7, D and E). Treatment with CD38i also resulted in an increase of PGC-1α, which was reversible after combined treatment with Ex527 (Figure 7F). In line with the previous results, CD38i improved expression of ETC components, i.e., ATP5a, SDHA, and cytochrome c, in noncontrollers (Figure 7, G and H). However, CD38i did not impact CPT1a expression (Supplemental Figure 7C).

To evaluate if the CD38i effects on mitochondrial metabolism and glycolysis resulted in altered effector functionality, we assessed cytokine production of CD38i-treated pp65_495–503_–CD8^+^ T cells after stimulation. We detected an increase in IFN-γ producers and triple IFN-γ/TNF/IL-2 producers (Figure 7, I and J, and Supplemental Figure 7D) in CMV-specific CD8^+^ T cells from noncontrollers, whereas no effects were observed in controllers. Moreover, addition of CD38i to etomoxir treatment reinvigorated the IFN-γ production in noncontrollers, indicating that FAO blockade is reversible after additional treatment with CD38i, which may relate to increased levels of NAD^+^, which boost glycolysis (Figure 7K). To further assess this hypothesis, we preincubated PBMCs of noncontrollers for 16 hours in the presence of CD38i (or vehicle) and stimulated them subsequently with pp65_495–503_ peptide for 4 hours until SCENITH analysis was performed. Strikingly, we observed an increased glycolytic capacity and decreased mitochondrial dependence on activated CD8^+^ T cells after CD38i treatment (Figure 7L). In addition, we performed SCENITH analysis on unstimulated pp65_495–503_ CD8^+^ T cells of noncontrollers and observed an even stronger effect on glycolytic capacity and mitochondrial dependence after CD38i incubation (Supplemental Figure 7E). Together, these data show that blocking the hydrolase activity of CD38 results in restoration of the metabolic and effector functionality of CMV-specific T cells in KTR CMV noncontrollers.

### Mouse model of CMV infection confirms an increased CD38 expression on CMV-specific CD8^+^ T cells and association with metabolic alterations.

**Figure 8:**
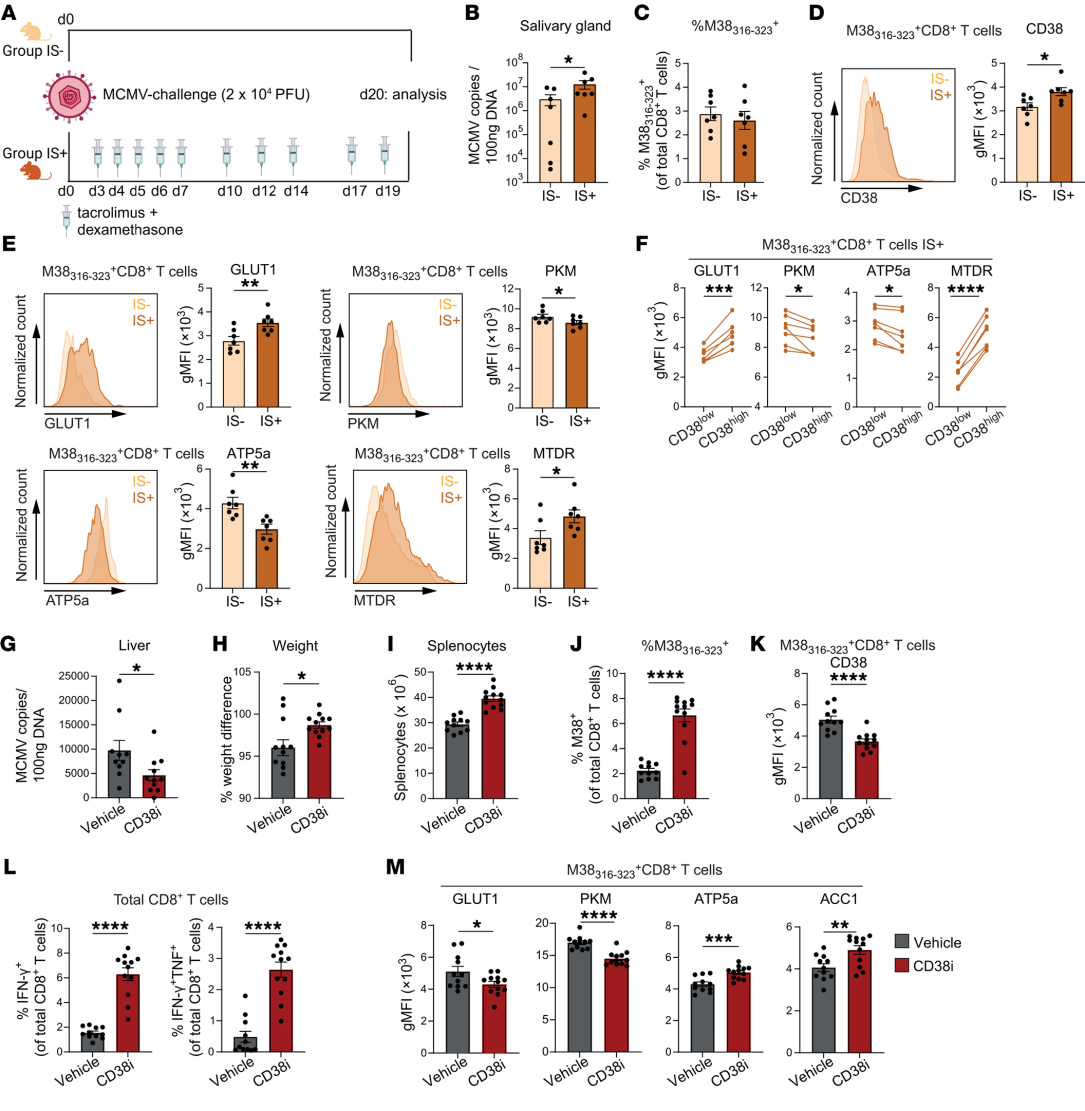
Mouse model of CMV infection confirms an increased CD38 expression on CMV-specific CD8^+^ T cells and association with metabolic alterations. (**A**–**F**) C57BL/6 mice were infected with MCMV-Smith (2 × 10^4^ PFU). Mice received immunosuppression (IS) medication (tacrolimus + dexamethasone) starting on day 3 after MCMV challenge (IS^+^, *n* = 7) or were untreated (IS^–^, *n* = 7). On day 20 salivary glands and spleens were isolated for determination of viral load and metabolic state of MCMV-specific CD8^+^ T cells, respectively. (**A**) Experimental setup. Created with biorender.com. (**B**) MCMV load in salivary glands. (**C**) Frequency of M38_316–323_–specific CD8^+^ T cells. (**D**) CD38 expression on M38_316–323_–specific CD8^+^ T cells. (**E**) GLUT1, PKM, ATP5a, and MTDR levels of M38_316–323_–specific CD8^+^ T cells from IS^–^ and IS^+^ mice. (**F**) GLUT1, PKM, ATP5a, and MTDR levels in CD38^lo^ and CD38^hi^ M38_316–323_–specific CD8^+^ T cells from IS^+^ mice. (**G**–**M**) C57BL/6 mice were infected with MCMV-Smith and received immunosuppression as above. On day 20, 1 group received CD38i twice daily for 7 consecutive days (*n* = 12) while the another (control) group received vehicle (*n* = 11). On day 27 livers and spleens were isolated for determination of viral load and metabolic state of MCMV-specific CD8^+^ T cells, respectively. (**G**) MCMV load in livers. (**H**) Weight difference before and after CD38i treatment. (**I**) Total splenocyte count. (**J**) Frequency of M38_316–323_–specific cells. (**K**) CD38 expression on M38_316–323_–specific CD8^+^ T cells. (**L**) Percentage IFN-γ^+^ and IFN-γ^+^TNF^+^ cells of total CD8^+^ T cells after stimulation for 5 hours with peptide. (**M**) Metabolic protein expression of M38_316–323_–specific CD8^+^ T cells. Data are presented as mean ± SEM. Each symbol represents an individual. Statistical analysis by 2-sided *t* test, paired *t* test, or Mann-Whitney *U* test (viral load). *P < 0.05; **P < 0.01; ***P < 0.001; ****P < 0.0001.

To recapitulate the observed association between CMV replication, increased CD38 expression and its effect on cellular metabolism of CMV-specific CD8^+^ T cells, we performed a controlled in vivo study (Figure 8A). WT mice received 2 × 10^4^ PFU MCMV, and to mimic immunosuppression as occurs in KTRs, we treated a subgroup of mice with tacrolimus and dexamethasone (38). MCMV load in the salivary glands at day 20 was elevated in mice receiving immunosuppression (Figure 8B). The splenic M38_316–323_–specific CD8^+^ T cells, which reflect the inflationary pp65-specific CD8^+^ T cells in humans (39), were unchanged in frequencies, but these cells displayed increased KLRG1 and CD44 expression, which is in agreement with the higher viral load following immunosuppression (Figure 8C and Supplemental Figure 8A). As in the human setting, we detected elevated CD38 expression on the CMV-specific CD8^+^ T cells in the immunosuppressed host, accompanied by an increased expression of GLUT1 and a decreased expression of PKM (Figure 8, D and E). In accordance, expression of ATP5a was decreased in the immunosuppressed mice and the mitochondrial mass and CPT1a was elevated (Figure 8E and Supplemental Figure 8B). Next, we evaluated the metabolic differences between CD38^lo^ and CD38^hi^ M38_316–323_–specific CD8^+^ T cells in the immunosuppressed group (Supplemental Figure 8C). We observed an increase in GLUT1, mitochondrial mass, and CPT1a in CD38^hi^ cells, whereas the expression of PKM and ATP5a was lower compared with CD38^lo^ cells, findings that were in line with those in KTRs (Figure 8F and Supplemental Figure 8D). Analysis of the total CD8^+^ T cells of naive mice treated with the same immunosuppressive protocol (tacrolimus and dexamethasone) revealed a strong decrease in the number of total splenic leucocytes in treated mice (Supplemental Figure 8E). However, only slight differences in the metabolic markers between uninfected mice with or without immunosuppression were detected (Supplemental Figure 8F), indicating a marginal direct impact of the immunosuppressive regimen on the metabolism of CD8^+^ T cells with the used dosages.

Given the reinvigorating effect on the metabolism and effector function of human CMV-specific CD8^+^ T cells after CD38i treatment ex vivo, we challenged mice with 2 × 10^4^ PFU MCMV-Smith and treated them with the same immunosuppressive regime as described above (Figure 8A). On day 20, mice received either CD38i twice daily or vehicle (36) while immunosuppressive treatment continued. After 1 week of CD38i treatment mice were sacrificed for further analysis. Interestingly, whereas untreated mice lost weight the CD38i-treated mice did not lose weight. This was accompanied by a higher number of splenocytes and a lower MCMV load in the liver of CD38i-treated mice compared with vehicle-treated mice, indicating a beneficial clinical effect of CD38i treatment within 1 week (Figure 8, G–I). Accordingly, the percentage of M38_316–323_–specific CD8^+^ T cells was increased in the CD38i group (Figure 8J), and the CD38 expression on the M38_316–323_–specific CD8^+^ T cells was lower compared with their counterparts in the vehicle group (Figure 8K). Importantly, the effector cytokine production of the M38_316–323_–specific CD8^+^ T cells increased after CD38i treatment (Figure 8L and Supplemental Figure 8G). Although expression of both GLUT1 and PKM was lower in M38_316–323_–specific CD8^+^ T cells of CD38i-treated mice, the expression of ATP5a, ACC1, and CPT1a was increased (Figure 8M and Supplemental Figure 8H). The latter relates to an increased capacity of mitochondrial fatty acid breakdown and mitochondrial respiration accompanied by an increase of anabolic fatty acid synthesis. Together, these data show that CD38 expression on CMV-specific CD8^+^ T cells increases in mice enduring CMV infection under immunosuppressive conditions, which associates with an increased mitochondrial mass and reduced expression of ATP5a. Inhibiting the NADase activity of CD38 leads to an expansion of CMV-specific CD8^+^ T cells with increased effector cytokine production and improved viral control, and this relates to improved mitochondrial metabolism.

## Discussion

Despite the high clinical relevance of CMV-associated morbidity and mortality in patients who are immunocompromised, the exact immunological mechanisms enabling sufficient viral control in these patients are still unidentified. Whereas certain patients, especially in transplantation medicine, develop severe and recurring CMV viremia with clinical symptoms (noncontrollers), other patients are in immunological control of the virus (controllers). Here, we provide in-depth metabolic profiling of CMV-specific CD8^+^ T cells at single-cell resolution in KTR noncontrollers, controllers, and HCs. We showed that CMV-specific CD8^+^ T cells in noncontrollers exhibit impaired glycolytic and mitochondrial capacity. The metabolic alterations in these noncontrollers are driven by an increased and sustained expression of the NADase CD38 on virus-reactive CD8^+^ T cells, and these findings were corroborated in a controlled in vivo MCMV infection model. Moreover, the impaired cytokine production of the CMV-specific CD8^+^ T cells in noncontrollers was improved by targeted inhibition of the CD38 hydrolase activity.

Metabolic dysfunction has been shown for exhausted CD8^+^ T cells in chronic HBV infection (18, 22), HIV infection (40) and LCMV infection (41). In these chronic viral infections, exhausted CD8^+^ T cells are marked by an increased expression of PD-1 and other inhibitory receptors that are only minimally expressed on CMV-specific CD8^+^ T cells (39). Mitochondrial impairment with decreased OXPHOS (18, 22, 40, 41), increased mitochondrial mass, and increased ROS levels seems a key dysfunctionality of exhausted CD8^+^ T cells (18, 40). Unbalanced ROS levels, which we also detected in CMV-specific CD8^+^ T cells of noncontrollers, leads to cellular impairment (18, 42). Moreover, CMV-specific CD8^+^ T cells of noncontrollers were marked by an increased mitochondrial mass, which has been attributed to defects in proteasome and autophagic lysosome-dependent protein turnover in dysfunctional T cells (18, 37). With respect to this, we detected decreased gene expression related to lysosomal function in noncontrollers, which might be a reason for the observed increase in mitochondrial mass. Nonetheless, the reported findings of mitochondrial dysfunction were mostly accompanied by a reduction of mitochondrial membrane potential and therefore an increased fraction of cells with depolarized mitochondria (18, 40). This contrasts with our results in CMV-specific CD8^+^ T cells, where an increased membrane potential was detected in noncontrollers. However, it has been described that chronic antigen stimulation can lead to mitochondrial hyperpolarization as shown in tumor-infiltrating lymphocytes in human renal cell carcinoma, also associated with increased ROS levels (43). Therefore, dependent on the context, mitochondrial depolarization, but also hyperpolarization of CD8^+^ T cells, can be considered as markers of mitochondrial dysfunction.

Data on glycolysis and FAO in the context of dysfunctional CD8^+^ T cells are less coherent in literature. Schurich et al. and Alrubayyi et al. reported an increased expression of GLUT1 in exhausted HBV- and HIV-specific CD8^+^ T cells, respectively (22, 40). Although expression levels of glycolytic enzymes were not investigated, it was hypothesized that exhausted T cells have an enhanced dependence on glycolysis to counteract mitochondrial dysfunction. Bengsch et al. investigated the metabolism of exhausted CD8^+^ T cells during LCMV-clone 13 infection. In contrast to the aforementioned studies, they detected an early reduction in glycolysis (41). These findings are supported by Patsoukis et al., who reported an inhibition of glycolysis and promotion of FAO in PD-1^+^CD4^+^ T cells (28). These alterations in metabolic pathways are in accordance with our results as we detected an increased expression of FAO components and decreased glycolytic enzyme expression in CMV-specific CD8^+^ T cells of noncontrollers.

CD38 is recognized as a marker for T cell activation and has previously been described as a marker on T cells responding to CMV replication in liver transplant recipients (44). More recently, the functional role has been linked to metabolism through its function as a major NADase. Correlations between increased CD38 expression on T cells and decreased intracellular NAD^+^ levels have been observed in multiple studies (30, 36, 45, 46). Moreover, it is described that NAD^+^ decline has a restraining effect on glycolysis as it is required in 2 enzymatic steps, and decreased SIRT1 activity negatively regulates the expression of glycolytic enzymes (47). A rise in CD38 expression and declining NAD^+^ levels are also directly coupled to decreased enzymes involved in glycolysis, including the M2 isoform of pyruvate kinase and antioxidative defense (46). Due to the limited number of antigen-specific CD8^+^ T cells in immunocompromised patients we were not able to directly measure NAD^+^ levels. Therefore, we used the NAD^+^/SIRT1/EZH2 axis as in indirect readout. However, SIRT1 is a promiscuous deacetylase that has multiple targets, and its inhibition is likely to affect CD8^+^ T cells on multiple levels.

Functional analysis revealed that IFN-γ production is strongly decreased after inhibition of glycolysis in controllers, whereas blockade of FAO only had a small effect. It is known that glycolysis is important for early ATP production after stimulation (48), and especially the production of IFN-γ by activated T cells is reliant on glycolysis (49). However, we observed a different pattern in noncontrollers, which were, compared with controllers, more affected by blockade of FAO than of glycolysis. In addition, we showed a strong dependence of translation on mitochondrial ATP generation in CMV-specific CD8^+^ T cells of noncontrollers in both a resting state and early after activation.

The usage of MHC class I tetramers enabled analysis of virus-specific CD8^+^ T cells at the single-cell level. Although we found comparable findings with pp65 and IE-1–specific CD8^+^ T cells, a limitation of this study is that we did not investigate responses against subdominant epitopes, which might be different between controllers and noncontrollers. In addition, we did not analyze other immune cells like CD4^+^ T cells or NK cells, which are also known to play an important role in controlling CMV infection.

A key finding of our study is the improved cytokine production of the CMV-specific CD8^+^ T cells of noncontrollers by inhibiting the NADase activity of CD38 with CD38i. Recently, CD38i has also been used to improve the functionality of HBV-specific CD8^+^ T cells (18, 42). Moreover, in patients with systemic lupus erythematodes (SLE) and increased susceptibility to infections, it was shown that CD38 overexpression in CD8^+^ T cells leads to mitochondrial dysfunctionality accompanied by restricted cytokine production, which could be improved by CD38i treatment. The authors were also able to show that CD38 inhibition in vivo had a beneficial effect on controlling LCMV infection in BXD2 lupus-prone mice (30, 37). In accordance, we showed that in vivo treatment of immunosuppressed MCMV-infected mice with CD38i led to a strong expansion and enhanced effector cytokine functionality of MCMV-specific CD8^+^ T cells, and this associated with no weight loss, improved viral control, and an increased mitochondrial metabolism. We did not observe signs of improved glycolytic capacity at the time of analysis, which may relate to the longitudinal treatment for a week, in which the viral-specific CD8^+^ T cells could have already adapted to a lower viral load. A complete deletion of CD38 in vivo, however, has been shown to improve proliferation and granzyme B production but impairs metabolism and survival of CD8^+^ T cells, indicating that moderate levels of CD38 are indispensable for CD8^+^ T cell metabolism (50).

In conclusion, our data provide an in-depth study on the metabolism of CMV-specific CD8^+^ T cells at different stages of CMV infection ranging from a controlled latent infection in HCs to an uncontrolled infection in patients who are immunocompromised. We identified metabolic dysregulations at the transcriptional, protein, and functional level that emerged during uncontrolled CMV infection. This was associated with elevated expression of CD38, where targeting of its NADase activity reinvigorated the metabolism and cytokine production of CMV-specific CD8^+^ T cells from noncontrollers, and this was recapitulated in an in vivo model. These findings could offer therapeutic opportunities for KTRs with uncontrolled CMV infection. However, an important note is that we did not focus on accessing the possible impact of a general CD38i treatment on other cell types, including a potential risk of provoking acute rejection by activating alloreactive T cells. We did not detect changes in the cytokine and metabolic profile in CMV-specific CD8^+^ T cells of controllers after CD38i treatment, indicating that its effect is closely related to increased CD38 expression on T cells. Further identifying the roles of critical metabolic modulators in T cells controlling viral infection could prove to be essential for development of more effective T cell–based immunotherapeutic strategies.

## Methods

### Sex as biological variable.

Our study examined men and women, and similar findings are reported for both sexes. Our study also examined male and female animals, and similar findings are reported for both sexes.

### Human study population.

The main cohort was included at the outpatient clinic of the Department of Nephrology, University Hospital Essen. Individuals with acute SARS infection were included during a cohort study at Leiden University Medical Center. For details see the Supplemental Methods.

### Animals.

C57BL/6J mice were obtained from Janvier labs (France). At the start of the experiments, mice were 7–10 weeks old. Animals were housed in individually ventilated cages under specific pathogen-free conditions at the animal facility at Leiden University Medical Center (LUMC).

### Sample processing and analysis.

Details of sample processing, gene expression profiling, flow cytometry, analyses, and reagents are included in the Supplemental Methods.

### Statistics.

Statistical analyses were performed using GraphPad Prism (version 8). Data were tested for normality using Shapiro-Wilk test. The specific tests used are indicated in the figure legends. Data between 2 groups were compared by *t* test or Mann-Whitney *U* test. Comparisons between more than 2 groups were done by 1-way ANOVA with Tukey’s test for multiple comparisons. When comparing multiple variables across 2 or more groups, 2-way ANOVA with Šidák’s test for multiple comparisons was performed. If comparing parameters within the same sample, a paired *t* test (2 groups) or a repeated-measures ANOVA with Geisser-Greenhouse correction (more than 2 groups) was used. Unless otherwise indicated, differences were not statistically significant. A *P* value under 0.05 was considered statistically significant.

### Study approval.

Studies on the main study cohort were approved by the local Ethics Committee of the University Hospital Essen, University of Duisburg-Essen, Germany (16-7229-BO). Before inclusion, all participants gave written informed consent. Individuals with acute SARS-CoV-2 infection were recruited through a cohort study, with ethical approval number NL77841.058.21. This study has been registered at clinicaltrials.gov under study number NCT06039527. All animal experiments were approved by the Animal Experiments Committee of LUMC and performed according to the recommendations and guidelines set by LUMC and by the Dutch Experiments on Animals Act (permit number AVD1160020186804).

### Data availability.

A Supporting Data Values file is available in accordance with *JCI* policy.

## Author contributions

NM, BW, and RA conceptualized the study. NM, GAH, BE, and RA designed the research studies and methodology. NM, FMB, SVD, FJVH, INP, ETIVDG, WV, TCVDS, JFDG, DMBV, XL, LVDS, and MJPW conducted experiments. NM and FMB analyzed data. KB and BW recruited patients. GAH, KLMCF, SHVDB, MJPW, SH, WH, SPJ, MG, and BE provided critical reagents, technical support, and patient samples. OW, APJDV, and AK provided resources. NM and RA wrote the manuscript. All authors reviewed, edited, and approved the final manuscript.

## Supplementary Material

Supplemental data

Supporting data values

## Version 1 07/02/2024

In-Press Preview

## Version 2 09/03/2024

Electronic publication

## Version 3 03/12/2025

Transposed definitions of “controllers” and “noncontrollers” corrected in first Results paragraph; tab labeling corrected in Supplemental Data Values file (6B and 6C).

## Version 4 03/13/2025

Error bar placement corrected in Figure 6B; label positioning adjusted in Figure 6D.
