# Supplementary material for: Inhibiting the NADase CD38 improves cytomegalovirus-specific CD8+ T cell functionality and metabolism
Source: J Clin Invest. 2024 Jul 2;134(17):e179561. doi: 10.1172/JCI179561 (PMC11364383; doi:10.1172/JCI179561)
Supplement: Supplemental data [file jci-134-179561-s009.pdf]

Supplemental Material

Inhibiting the NADase CD38 improves cytomegalovirus-specific CD8+ T cell functionality and metabolism

Nils Mülling[1,2], Felix M. Behr[1], Graham A. Heieis[3], Kristina Boss[2], Suzanne van Duikeren[1], Floortje J. van Haften[1], Iris N. Pardieck[1], Esmé T.I. van der Gracht[1], Ward Vleeshouwers[1], Tetje C. van der Sluis[1], J. Fréderique de Graaf[1], Dominique M.B. Veerkamp[1], Kees L.M.C. Franken[1], Xin Lei[1], Lukas van de Sand[4], Sjoerd H. van der Burg[5], Marij J. P. Welters[5], Sebastiaan Heidt[1], Wesley Huisman[3], Simon P. Jochems[3], Martin Giera[6], Oliver Witzke[4], Aiko P.J. de Vries[7], Andreas Kribben[2], Bart Everts[3], Benjamin Wilde[2], and Ramon Arens[1*]

[1] Department of Immunology, Leiden University Medical Center, 2333 ZA Leiden, The Netherlands
[2] Department of Nephrology, University Hospital Essen, University Duisburg-Essen, 45147 Essen, Germany
[3] Department of Parasitology, Leiden University Medical Center, 2333 ZA Leiden, The Netherlands
[4] Department of Infectious Diseases, University Hospital Essen, University Duisburg-Essen, 45147 Essen, Germany
[5] Department of Medical Oncology, Leiden University Medical Center, 2333 ZA Leiden, The Netherlands
[6] Center for Proteomics and Metabolomics, Leiden University Medical Center, 2333 ZA Leiden, The Netherlands
[7] Department of Internal Medicine, Leiden University Medical Center, 2333 ZA Leiden, The Netherlands

* Correspondence: R.Arens@lumc.nl

Contents:

Supplemental Figures 1-8

Supplemental Methods

Supplemental Tables 1-5

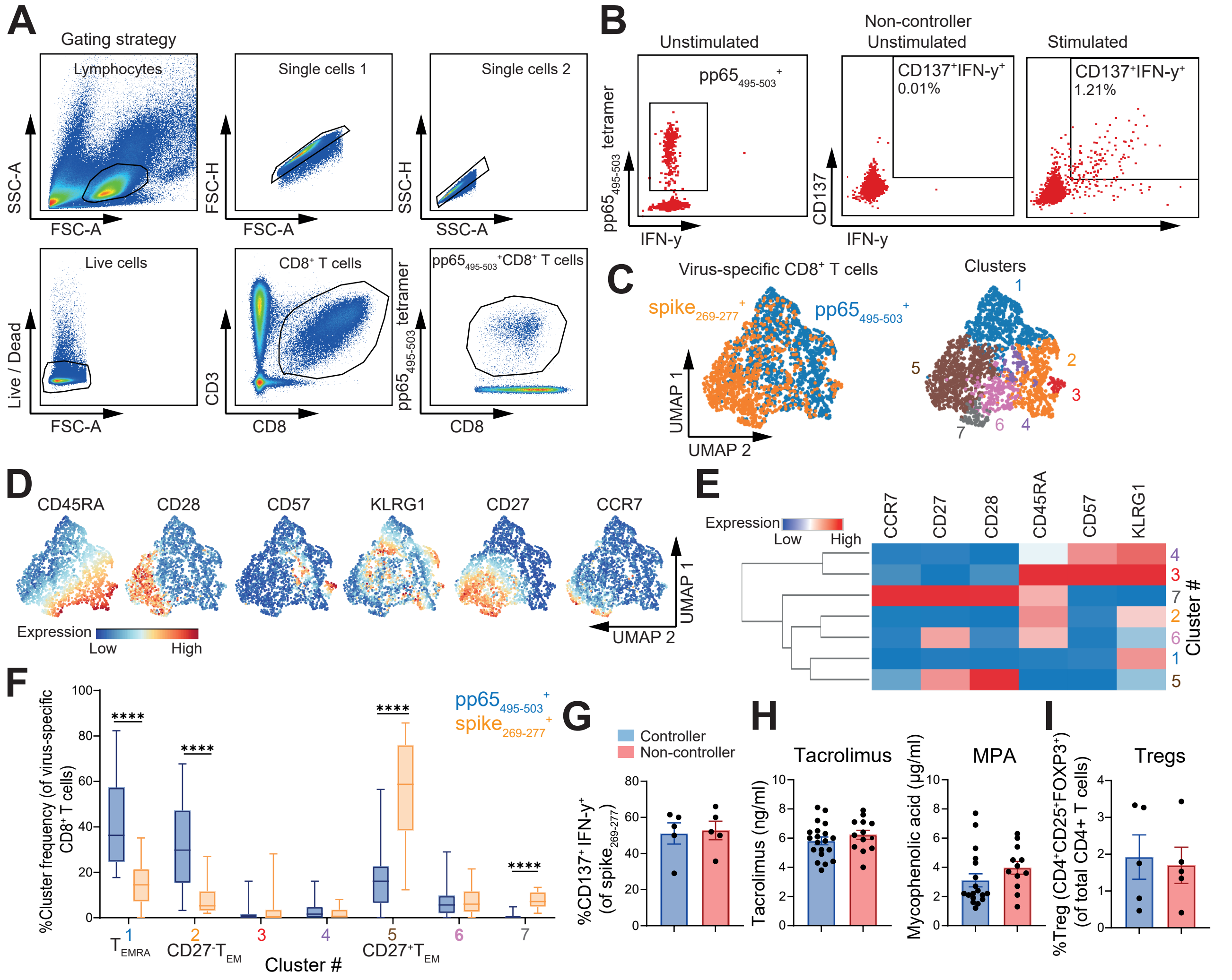

Supplemental Figure 1. Frequency and phenotype of CMV- and SARS-CoV-2-specific CD8⁺ T cells are unaffected by loss of viral control. (**A**) Representative flow cytometry gating strategy for the detection of MHC class I tetramer positive CD8⁺ T cells (shown for $pp65_{495\text{-}503}$). (**B**) After 20 hours stimulation with $pp65_{495\text{-}503}$ peptide, cytokine production was measured by intracellular cytokine staining. Representative flow cytometry plots from a non-controller. Plots show gated CD8⁺ T cells in unstimulated and stimulated condition. (**C**) Following UMAP analysis, FlowSOM consensus meta-clustering with 7 clusters was performed on 200 down-sampled $pp65_{495\text{-}503}$ and $spike_{269\text{-}277}$-specific CD8⁺ T cells from KTR (n = 24; 15 controller; 9 non-controller). Virus-specific T cell populations (left), overlay of the 7 FlowSOM clusters (right) on the UMAP. (**D**) Expression intensity of cell surface markers. (**E**) Hierarchically clustered heatmap of phenotypes of the clusters shown in (C). The indicated marker expression is shown per cluster as z-score of median signal intensity per channel. (**F**) Cluster frequencies of $pp65_{495\text{-}503}$ (blue) and $spike_{269\text{-}277}$-specific (orange) CD8⁺ T cells. (**G**) Percentage IFN-γ⁺CD137⁺ of $spike_{269\text{-}277}$-specific CD8⁺ T cells from controllers and non-controllers stimulated for 20 hours with peptide (n=5/group). (**H**) Blood levels of tacrolimus and mycophenolic acid (MPA) at the date of blood sampling. (**I**) Percentage of regulatory CD4⁺CD25⁺FOXP3⁺ T cells (Tregs) of total CD4⁺ T cells (n=5/group). Data are presented as mean ± SEM or as box-plot. Bounds of the boxes indicate upper and lower quartile, lines indicate median, whiskers indicate min and max. Each symbol in G-H represents an individual. Statistical analysis by two-sided student's t-test or two-way ANOVA with Sidak's test for multiple comparisons. *p<0.05; **p<0.01; ***p<0.001; ****p<0.0001.

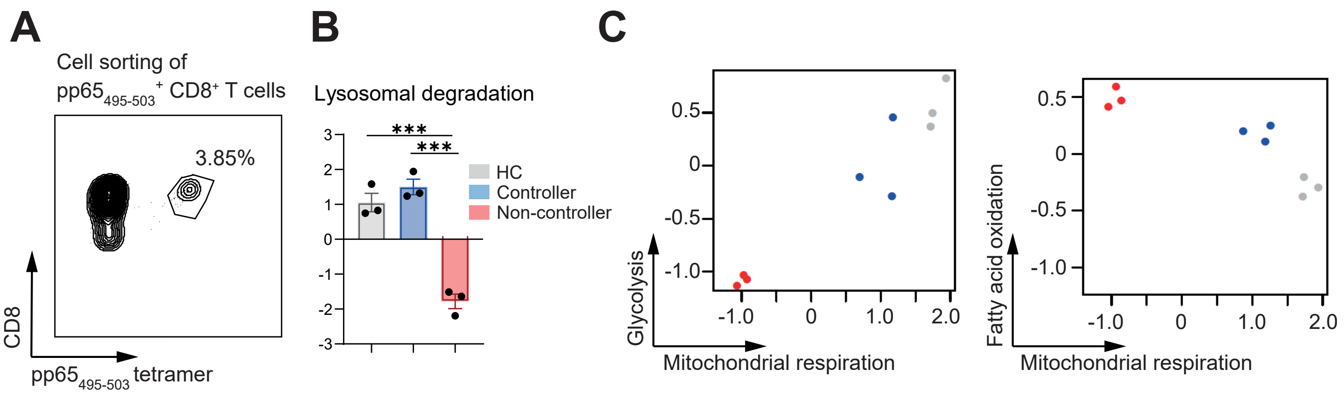

Supplemental Figure 2. Transcriptional analysis of metabolic pathways in CMV-specific CD8⁺ T cells. CMV pp65$_{495\text{-}503}$-specific CD8⁺ T cells (n=3 independent donors/group) were sorted from PBMCs and subjected to nCounter Metabolic Pathways. (**A**) Representative flow cytometry plot of sorting pp65$_{495\text{-}503}$$^{+}$CD8⁺ T cells. (**B**) Lysosomal degradation pathway score. Scores are presented as relative expression. (**C**) Signature scores plots of mitochondrial respiration with glycolysis and fatty acid oxidation, respectively. Scores are presented as relative expression. Data are presented as mean ± SEM. Each symbol represents an individual. Statistical analysis by one-way ANOVA with Tukey's test for multiple comparisons. *p<0.05; **p<0.01; ***p<0.001; ****p<0.0001.

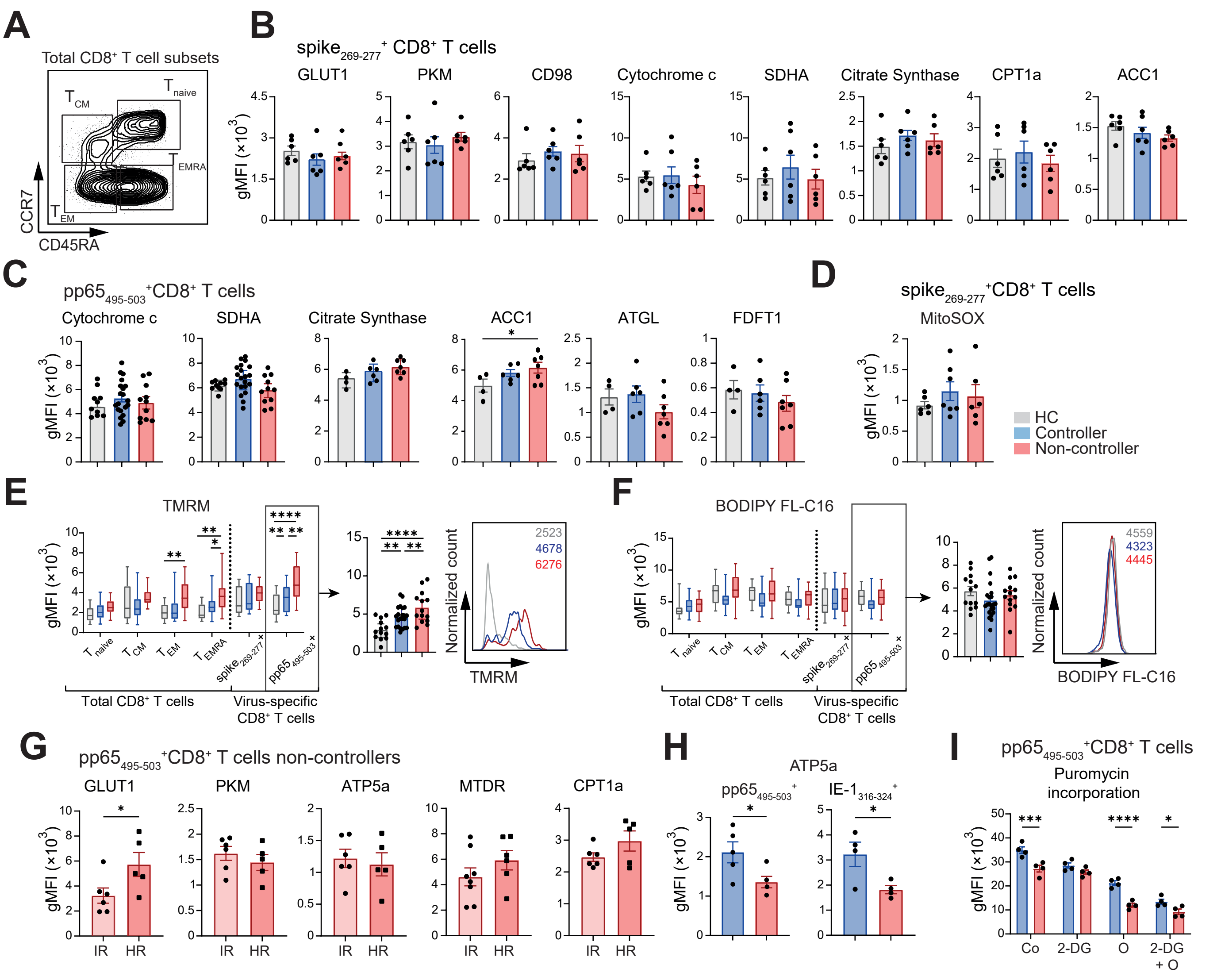

Supplemental Figure 3. CMV-specific CD8⁺ T cells of non-controllers exhibit restrained glycolysis and mitochondrial respiration while fatty acid metabolism is increased. (**A**) Representative flow cytometry plot for gating of total CD8⁺ T cell subsets according to their expression of the cell surface markers CCR7 and CD45RA. $T_{naïve}$ (naïve CD8⁺ T cells), T_{CM} (central-memory CD8⁺ T cells), T_{EM} (effector-memory CD8⁺ T cells), T_{EMRA} (effector-memory re-expressing RA CD8⁺ T cells). (**B**) Metabolic protein expression in $spike_{269\text{-}277}$-specific CD8⁺ T cells (n=6/group). (**C**) Metabolic protein expression in $pp65_{495\text{-}503}$-specific CD8⁺ T cells (n=4-22/group). (**D**) MitoSOX Red uptake in $spike_{269\text{-}277}$-specific CD8⁺ T cells (n=4-6/group). (**E**-**F**) TMRM and BODIPY FL-C16 uptake in different CD8⁺ T cell subsets (n=13-22/group). (**G**) Metabolic protein expression of $pp65_{495\text{-}503}$-specific CD8⁺ T cells from CMV intermediate-risk (IR) and high-risk (HR) non-controllers (combined data displayed in **Figure 3**). (**H**) Expression of ATP5a in $pp65_{495\text{-}503}$ and $IE\text{-}1_{316\text{-}324}$-specific CD8⁺ T cells within the same individuals ($IE\text{-}1_{316\text{-}324}$-specific cells could not be detected in one individual) (n=5/group). (**I**) SCENITH method on $pp65_{495\text{-}503}$-specific CD8⁺ T cells using puromycin incorporation following incubation with 2-deoxy-d-glucose (2-DG), oligomycin (O) or media controls (Co) (n=4/group). TMRM = Tetramethylrhodamine methyl ester. MTDR = MitoTracker Deep Red. Data are presented as mean ± SEM or as box-plot (bounds of the boxes indicate upper and lower quartile, line indicates median, whiskers indicate min and max). Each symbol represents an individual. Statistical analysis by two-sided student's t-test, paired t-test one-way ANOVA with Tukey's test for multiple comparisons, two-way ANOVA with Sidak's test for multiple comparisons. *p<0.05; **p< 0.01; ***p< 0.001; **** p<0.0001.

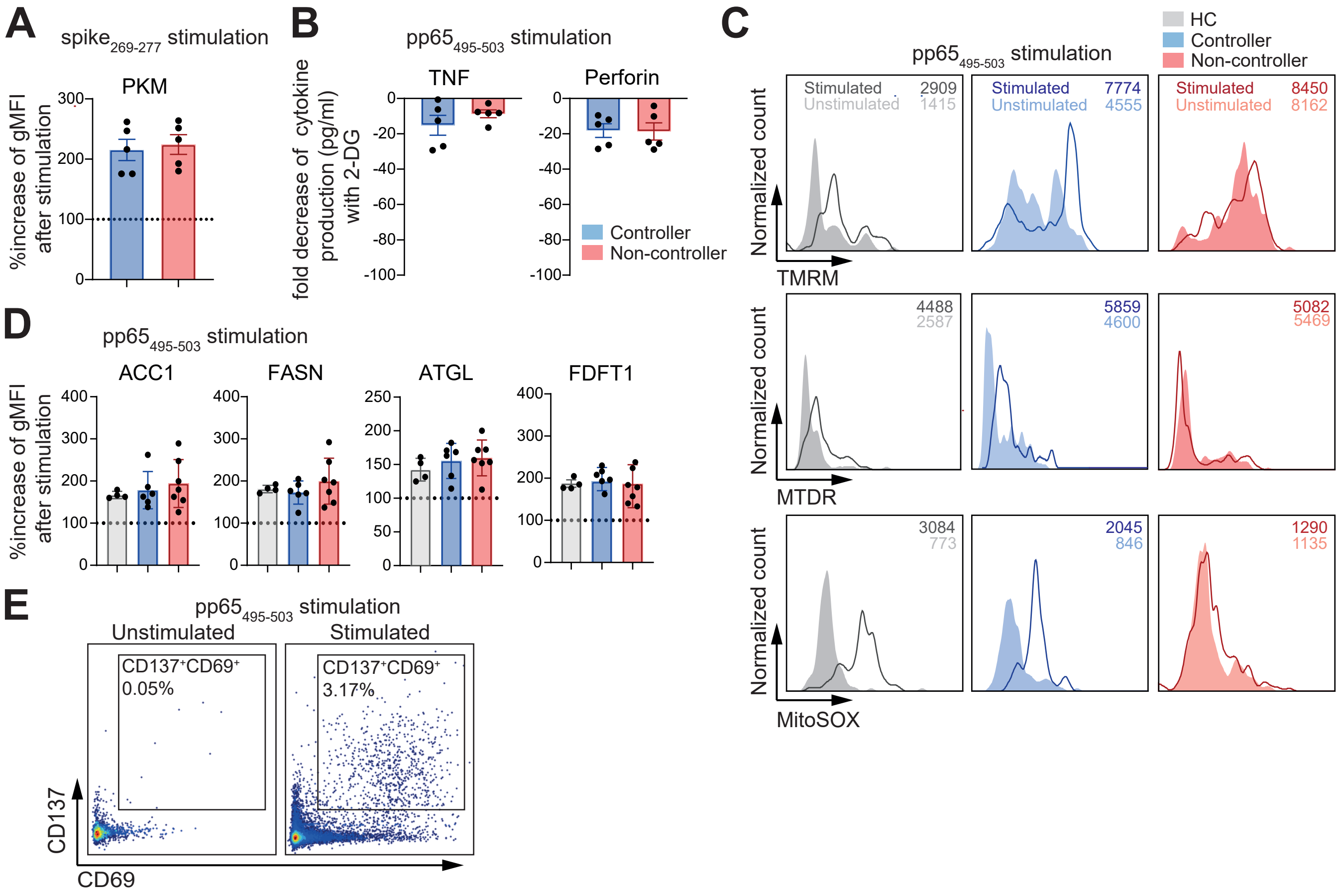

Supplemental Figure 4. Impaired metabolic responsiveness of CMV-specific CD8⁺ T cells in non-controllers. (**A**) Relative change (in %) of PKM1 expression of spike$_{269\text{-}277}$-specific CD8$^+$ T cells after long stimulation (6 days) compared to unstimulated condition (n=5/group). (**B-D**) PBMCs were stimulated with pp65$_{495\text{-}503}$ peptide for 20 hours (short stimulation). After stimulation, reactive cells were identified by CD137. (**B**) After short stimulation, supernatant was analyzed by Legendplex for the production of cytokines. The percentage decrease in cytokine concentration (pg/ml) after 2-DG treatment is shown (n=5/group). (**C**) Representative histograms depict the change in TMRM, MTDR and MitoSOX signal after short stimulation. Color indication: unstimulated, filled; stimulated, unfilled. Numbers are indicating geoMFI. (**D**) Relative change (in %) of ACC1, FASN, ATGL and FDFT1 expression of pp65$_{495\text{-}503}$-specific CD8$^+$ T cells after stimulation (6 days) compared to unstimulated condition (n= 4-7/group). (**E**) Representative flow cytometry plots show gating of CD137$^+$CD69$^+$CD8$^+$ T cells after 4 hours of stimulation with pp65$_{495\text{-}503}$ peptide. Data are presented as mean ± SEM. Each symbol in A, B, and D represents an individual.

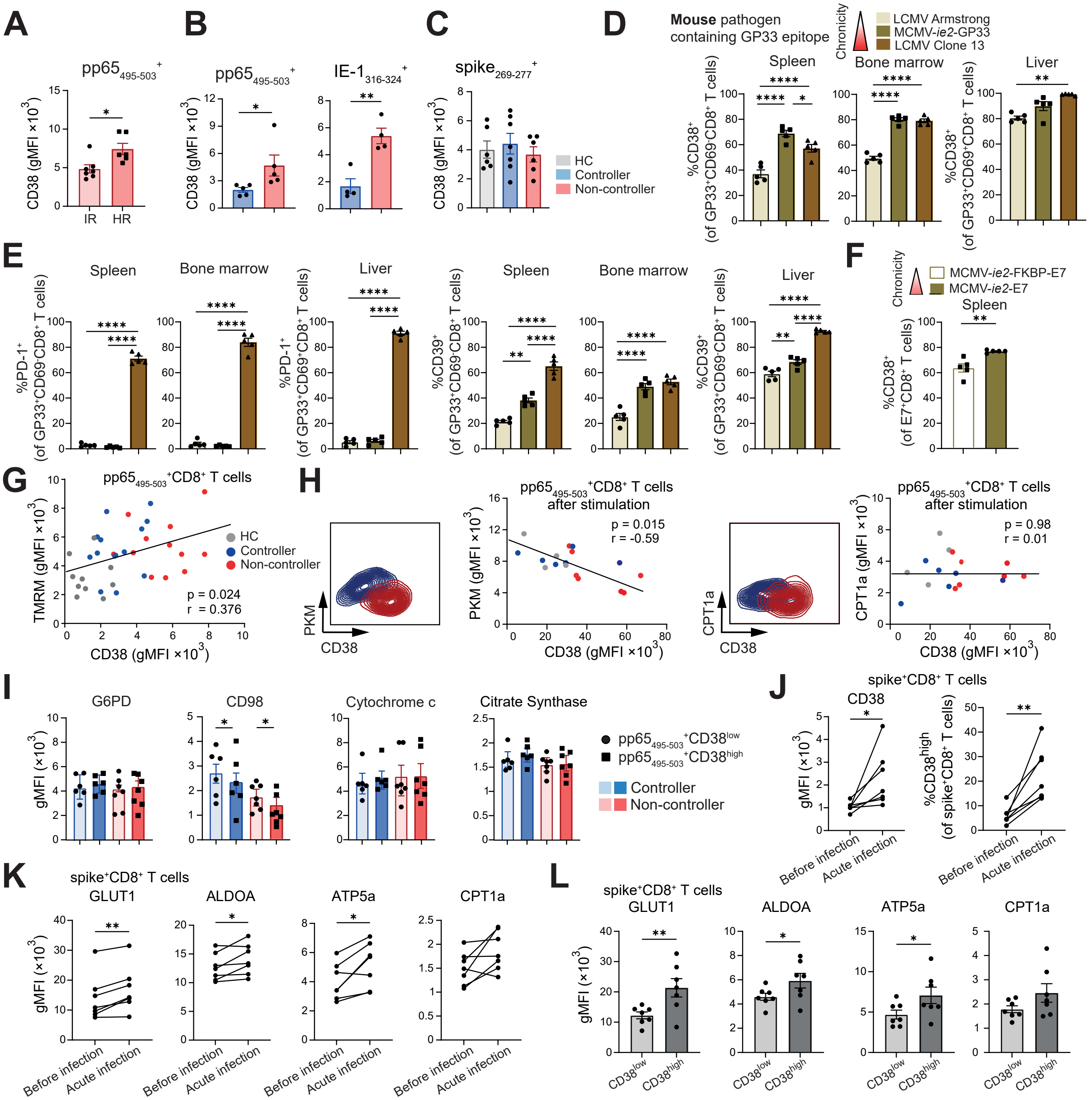

Supplemental Figure 5. CD38 expression is particularly elevated on active circulating CMV-specific CD8⁺ T cells and associates with metabolic dysfunction. (**A**) CD38 expression of pp65$_{495\text{-}503}$-specific CD8⁺ T cells from CMV intermediate-risk (IR) and high-risk (HR) non-controllers (combined data displayed in Figure 5A). (**B**) CD38 expression of pp65$_{495\text{-}503}$- and IE-1$_{316\text{-}324}$-specific CD8⁺ T cells within the same individuals (IE-1$_{316\text{-}324}$-specific cells could not be detected in one individual) (n=5/group). (**C**) CD38 expression of spike$_{269\text{-}277}$$^+$CD8⁺ T cells (n=6-7/group). (**D-E**) Percentage CD38⁺ (**D**), PD-1⁺ and CD39⁺ (**E**) GP$_{33\text{-}41}$-specific CD69⁻ and CD69⁺ CD8⁺ T cells in spleen, bone marrow and liver of C57BL/6 mice infected 50 days previously with LCMV-Armstrong, MCMV-*ie2*-GP33, or LCMV-clone 13. (**F**) Percentage CD38⁺ E7$_{49\text{-}57}$-specific CD69⁻ CD8⁺ T cells in spleen of C57BL/6 mice infected 50 days previously with MCMV-*ie2*-FKBP-E7 or MCMV-*ie2*-E7. (**G**) Correlation between the expression of CD38 and TMRM of pp65$_{495\text{-}503}$-specific CD8⁺ T cells (n=10-13/group). (**H**) PBMCs were stimulated for 6 days with pp65$_{495\text{-}503}$ peptide. Representative contour plots and correlation plots show the expression of PKM versus CD38 and CPT1a versus CD38 of pp65$_{495\text{-}503}$$^+$CD8⁺ T cells (n=4-7/group). (**I**) Metabolic protein expression of CD38^{low} and CD38^{high} pp65$_{495\text{-}503}$-specific CD8⁺ T cells (n=6-7/group). (**J-K**) Individuals (n=7) from a cohort study (no transplant patients) with acute SARS-CoV-2 infection were sampled before and within 7 days after symptom onset. (**J**) CD38 expression and percentage CD38^{high} of spike-specific CD8⁺ T cells in individuals before and during acute infection with SARS-CoV-2. (**K**) Metabolic protein expression of spike-specific CD8⁺ T cells in individuals before and during acute infection with SARS-CoV-2. (**L**) Metabolic protein expression of CD38^{low} and CD38^{high} spike-specific CD8⁺ T cells of individuals during acute infection with SARS-CoV-2. Data are presented as mean ± SEM. Each symbol represents an individual. Statistical analysis by one-way ANOVA with Tukey's test for multiple comparisons, two-sided student's t-test, paired t-test or Pearson correlation. *p<0.05; **p<0.01; ***p<0.001; ****p<0.0001.

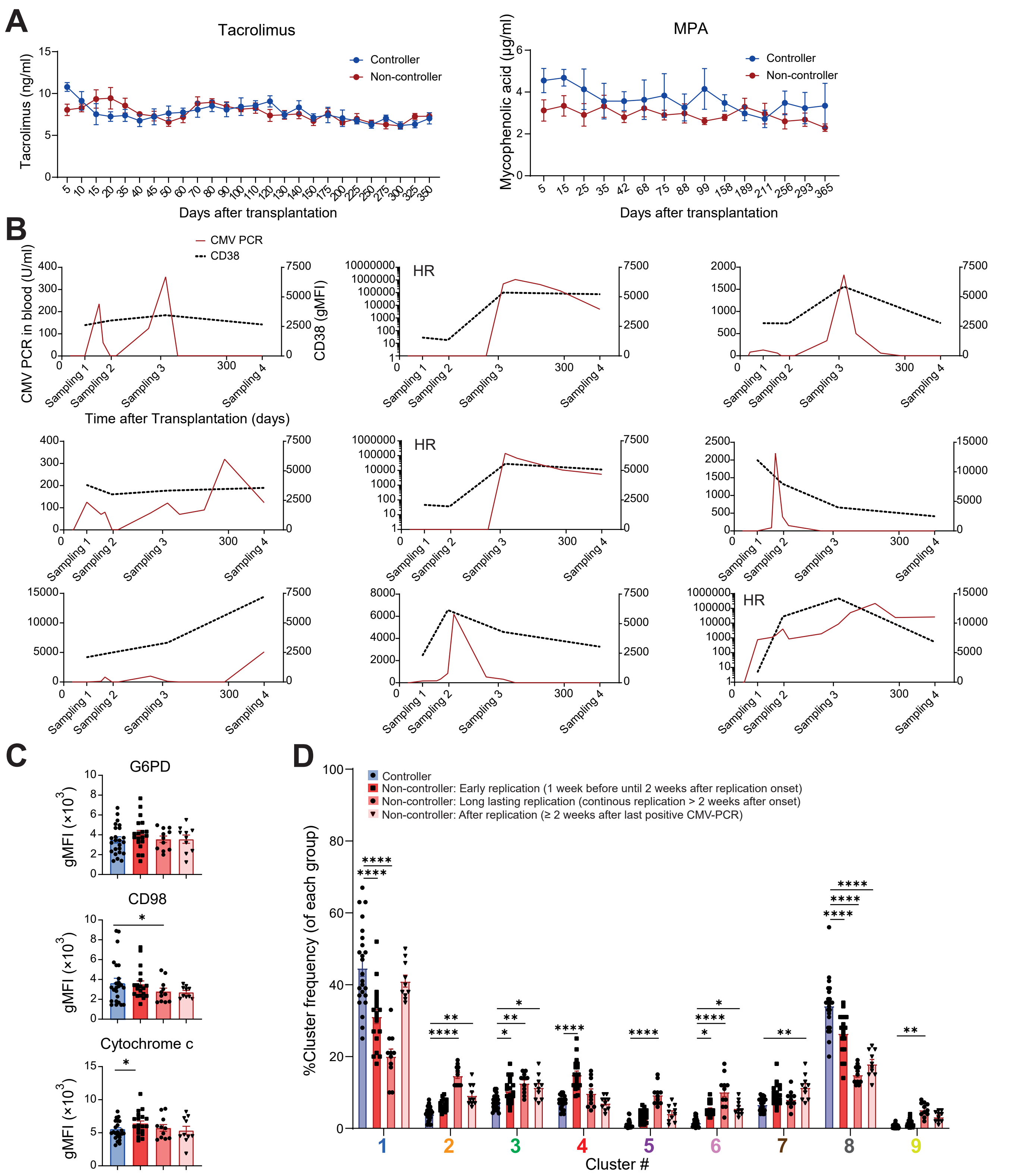

Supplemental Figure 6. Elevated expression of CD38 on CMV-specific CD8⁺ T cells during viral persistence gradually re-wires cellular metabolism. An independent cohort of 16 patients was longitudinally analyzed after kidney transplantation. Blood samples for analysis of $pp65_{495\text{-}503}$-specific $CD8^+$ T cells were taken at 6 weeks, 3 months, 6 months and 12 months after transplantation. CMV-replication in blood was continuously assessed for about 18 months. With respect to replication onset, the cohort was divided in patients without CMV replication (controller, n=6) and patients with CMV replication (non-controller, n=10). (**A**) Blood levels of tacrolimus and mycophenolic acid (MPA) over time. (**B**) Graphs show the CMV load in blood (left y-axis) and the expression of CD38 (right y-axis) over time in each non-controller (except the representative example shown in **Figure 6A**). Sampling 1 = analysis after 6 weeks, Sampling 2 = analysis after 3 months, Sampling 3 = analysis after 6 months, Sampling 4 = analysis after 12 months. HR = CMV high-risk patients. (**C**) Metabolic protein expression of $pp65_{495\text{-}503}$-specific $CD8^+$ T cells compared between groups. (**D**) Frequencies (%) of clusters (depicted in **Figure 6I-6L**) of $pp65_{495\text{-}503}$-specific $CD8^+$ T cells from each group. Data are presented as mean ± SEM. Each symbol represents an individual. Statistical analysis by one-way ANOVA with Tukey's test for multiple comparisons or two-way ANOVA with Sidak's test for multiple comparisons. *p<0.05; **p<0.01; ***p<0.001; ****p<0.0001.

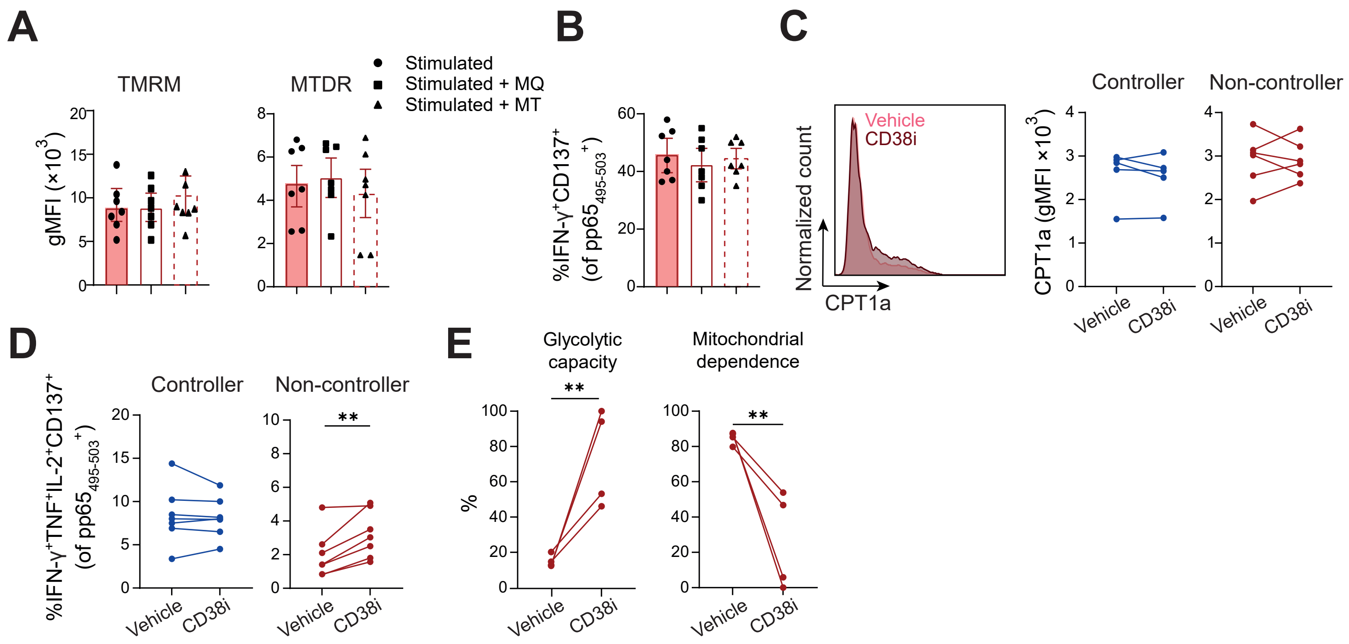

Supplemental Figure 7. CD38 inhibition restores metabolic dysregulation and improves functionality of CMV-specific CD8⁺ T cells in non-controllers. (**A**) PBMCs were stimulated with $pp65_{495\text{-}503}$ peptide for 20 hours, and subsequently MTDR and TMRM uptake was quantified and compared between stimulated, stimulated + Mitoquinone (MQ), and stimulated + MitoTEMPO (MT) (n=7 non-controllers). (**B**) After 20 h peptide stimulation, IFN-γ production was measured by intracellular cytokine staining and compared between stimulated, stimulated + MQ, stimulated + MT (n=7 non-controllers). (**C**-**D**) PBMCs from non-controllers were stimulated for 6 days with $pp65_{495\text{-}503}$ peptide in presence of vehicle or CD38i. (**C**) Representative histogram of CPT1a expression. Graphs display the change of CPT1a expression after CD38i treatment (n = 5-6/group). (**D**) Percentage IFN-γ^+TNF$^+$IL-2$^+$CD137$^+$ cells of $pp65_{495\text{-}503}$-specific CD8$^+$ T cells after stimulation for 20 hours with $pp65_{495\text{-}503}$ peptide in presence or absence of CD38i (n=7/group). (**E**) PBMCs were cultured in presence of CD38i or vehicle. After 16 hours, SCENITH was performed on $pp65_{495\text{-}503}$-specific CD8$^+$ T cells (n=4 non-controllers). Data are presented as mean ± SEM. Each symbol represents an individual. Statistical analysis by one-way ANOVA with Tukey's test for multiple comparisons or paired t-test. *p<0.05; **p<0.01; ***p<0.001.

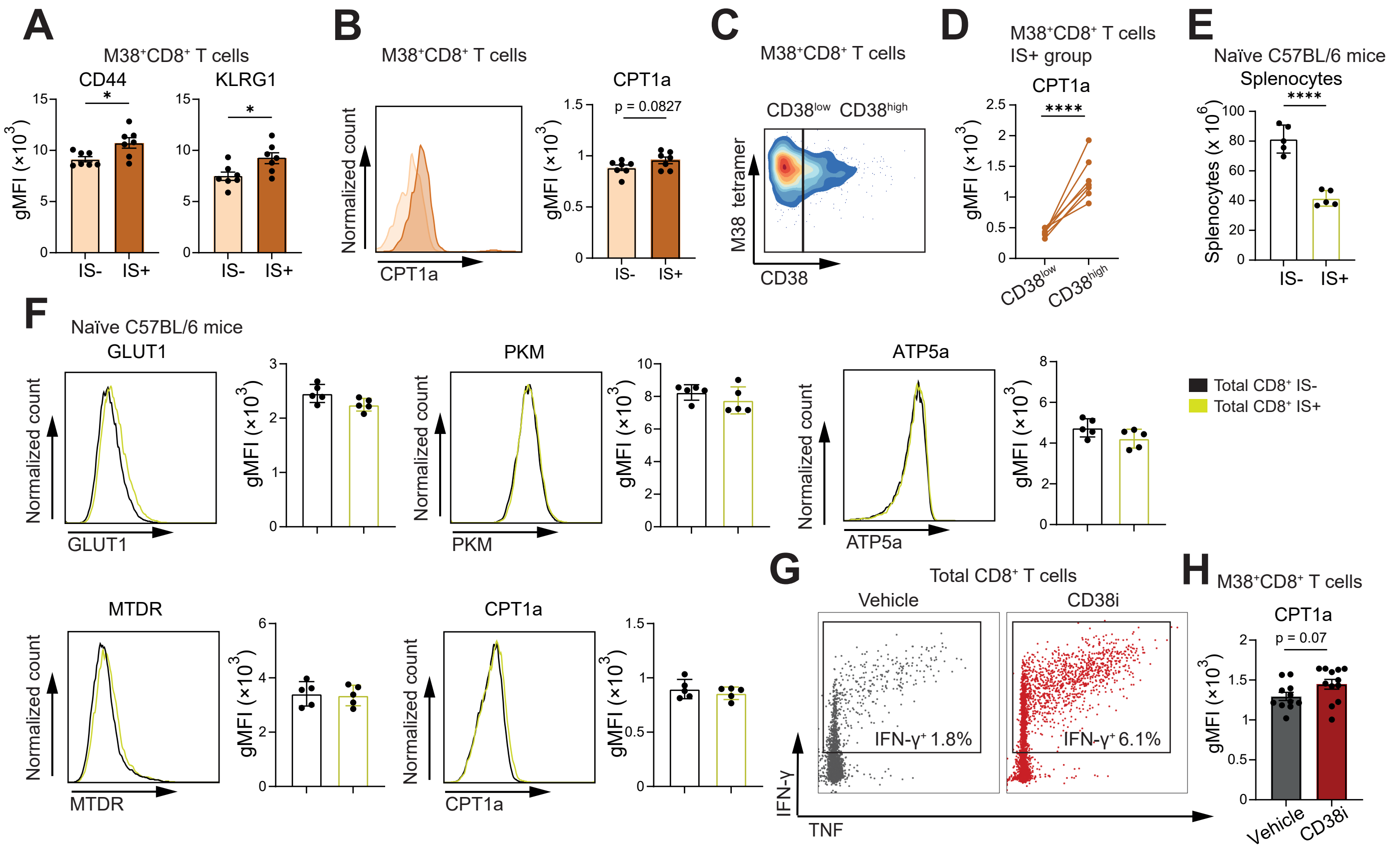

Supplemental Figure 8. Immunosuppressive medication of naïve mice does not affect the metabolism of CD8$^+$ T cells but leads to leucopenia. (**A-D**) C57BL/6 mice were infected with MCMV-Smith (2×10^4 PFU) and received either immunosuppressive medication (IS+, n=7) as shown in **Figure 8A** or not (IS-, n=7). On day 20 spleens were isolated for determination of the metabolic state of MCMV-specific CD8$^+$ T cells. (**A**) Expression of CD44 and KLRG1 on M38$_{316\text{-}323}$-specific CD8$^+$ T cells. (**B**) Representative staining and quantification of CPT1a expression of M38$_{316\text{-}323}$-specific CD8$^+$ T cells from IS- and IS+ mice. (**C**) Gating strategy to discriminate between CD38^{low} and CD38^{high} M38$_{316\text{-}323}$-specific CD8$^+$ T cells. (**D**) CPT1a expression in CD38^{low} and CD38^{high} M38$_{316\text{-}323}$-specific CD8$^+$ T cells from IS+ mice. (**E-F**) Naïve C57BL/6 mice received either immunosuppressive medication (IS+, n=5) or not (IS-, n=5) according to the same treatment schedule as shown in **Figure 8A**. (**E**) Total splenocyte count. (**F**) Metabolic protein expression and MTDR staining of total CD8$^+$ T cells. (**G-H**) C57BL/6 mice were infected with MCMV-Smith and received immunosuppression as above. On day 20 the treatment group received CD38i twice daily for 7 consecutive days (n=12). The control group received vehicle (n=11). On day 27 spleens were isolated for determination of the cytokine production and metabolic state of MCMV-specific CD8$^+$ T cells. (**G**) Representative flow cytometry plots showing the expression of IFN-γ and TNF after 5 hours of M38$_{316\text{-}323}$ peptide stimulation. (**H**) CPT1a expression of M38$_{316\text{-}323}$-specific CD8$^+$ T cells. Data are presented as mean ± SEM. Each symbol represents an individual. Statistical analysis by two-sided t-test or paired t-test. *$p<0.05$; **$p<0.01$; ***$p<0.001$; ****$p<0.0001$.

Supplemental Methods

Kidney transplant recipients and healthy control group

A total of 52 kidney transplant recipients (KTRs) and 13 healthy controls (HC) were included. Main study population: Patients with CMV high- or intermediate-risk category and anti-CMV IgG positive healthy volunteers were included (**Supplemental Table 1**). All individuals were positive for HLA-A*02 and received at least two doses of mRNA vaccines against SARS-CoV-2 (bnt162b2 or mrna-1273). An interval of at least 14 days to last vaccine dose was required. Individuals with an acute, PCR-proofed SARS-CoV-2-infection were excluded. Further exclusion criteria for patients were: kidney transplantation within last 3 months, a rejection therapy within last 3 months and an eGFR <15ml/min/m^2 (**Supplemental Table 2**). Most of the patients received triple immunosuppressive therapy with tacrolimus, mycophenolate, and prednisone (**Supplemental Table 1**). Kidney transplant recipients were further divided into two groups, based on CMV-PCR results (threshold defined as a result > 65 IU/ml) and clinical data: 1. KTR CMV non-controllers: CMV-replication (> 300 IU/ml blood) without spontaneous clearance by the host and/or symptomatic infection (CMV syndrome or disease) in last 6 months. Requirement of anti-viral treatment to stop viral replication. 2. KTR CMV controllers: No CMV-replication or asymptomatic, low level (<300 IU/ml blood) CMV replication in last 6 months. No requirement of anti-viral treatment to stop viral replication.

For the longitudinal analysis referring to **Figure 6** and **Supplemental Figure 6**, a total of 16 KTRs (6 controllers, 10 non-controllers) were studied for 15 months after transplantation. Blood was taken at 6 weeks, 3 months, 6 months, and 12 months after transplantation. PBMCs were isolated and cryopreserved. Samples were thawed and analyzed on one day. The patients were clinically monitored and CMV replication in blood was assessed 10-15 times/patient during study period. All patients were HLA-A*02-positive and at CMV intermediate-risk (controllers n=6; non-controllers n=7) or high-risk constellation (controllers n=0; non-controllers n=3). Immunosuppression was equal for all longitudinally analyzed patients: induction therapy with basiliximab; maintenance therapy with tacrolimus, prednisone, and mycophenolate. No rejection therapy occurred during study period.

Individuals with acute SARS-CoV-2 infection

Individuals (n=7) were recruited through a cohort study, with ethical approval number NL77841.058.21. This study has been registered at clinicaltrials.gov under study number: NCT06039527. Individuals aged 18-30 years old, or over 65 years old were included. Baseline samples, including PBMCs, were collected from all participants. Additional PBMCs samples were collected from participants if they experienced symptoms of respiratory infection within 3 months after baseline sampling. These samples were collected within 7 days of symptom onset and participants with a positive result for SARS-CoV-2 (PCR or self-test) were included for this analysis. For the present study frozen PBMCs of n=7 study participants (one sample before and one sample during acute infection/patient) were used. All individuals received SARS-CoV-2 vaccination before first blood sampling.

Sample processing

Blood from patients and healthy donors was drawn at the outpatient clinic of the Department of Nephrology, University Hospital Essen. Blood from patients was drawn before the intake of immunosuppressive medication. Heparinized blood was shipped per overnight-express at room

temperature to the Department of Immunology, Leiden University Medical Center. Immediately after arrival (within 24 hours after blood donation), peripheral blood mononuclear cells were isolated by Ficoll-Hypaque (GE Healthcare) density gradient centrifugation and cryopreserved in liquid nitrogen until the day of analysis. For analysis, PBMCs were thawed at 37°C, and washed with Iscove's Modified Dulbecco's Medium (IMDM, Capricorn Scientific or Lonza) supplemented with 8% FCS (Bodinco), 100 U/ml penicillin (Gibco), 100 U/ml streptomycin (Gibco), 2mM L-glutamine (Gibco). Next, PBMCs were rested in IMDM containing 30 µg/ml DNAse I (Merck) at 37°C for 2 hours to recover before subsequent procedures.

MHC class I tetramers and synthetic peptides

MHC class I tetramers were generated in-house and labelled with either APC or PE (**Supplemental Table 3**). HLA-A*02-restricted HCMV $pp65_{495\text{-}503}$ (NLVPMVATV), HLA-A*02-restricted HCMV $IE\text{-}1_{316\text{-}324}$ (VLEETSVML), and HLA-A*02-restricted SARS-CoV-2 $spike_{269\text{-}277}$ (YLQPRTFLL) peptide/tetramer complexes were used to identify human viral-specific $CD8^+$ T cells. To identify MCMV-specific $CD8^+$ T cells in mice, H2-Kb-restricted $M38_{316\text{-}323}$ (SSPPMFRV) peptide/tetramer complexes were used. For stimulation of HCMV-specific $CD8^+$ T cells the following 9-mer peptide including the HLA-A*02-restricted HCMV $pp65_{495\text{-}503}$ (NLVPMVATV) epitope was used. For stimulation of SARS-CoV-2 specific $CD8^+$ T cells the following 9-mer peptide including the HLA-A*02-restricted $spike_{269\text{-}277}$ (YLQPRTFLL) epitope was used. MHC class I tetramer mix containing multiple epitopes restricted to different HLA types was used to analyze blood samples from individuals with acute SARS-CoV-2 infection (**Supplemental Table 5**).

Flow cytometry

Viability, cell surface and tetramer staining

To assess viability, dead cells were excluded by staining with Zombie NIR (BioLegend) or Live Dead Fixable Blue (ThermoFisher) in PBS for 15 minutes at room temperature. Tetramer staining was performed in staining buffer (PBS + 1% FCS) for 30 minutes at 4 °C. For cell surface staining, cells were incubated with fluorescently-labeled antibodies for 30 minutes at 4 °C. All used antibodies and dead cell exclusion dyes are indicated in **Supplemental Table 4**.

Mitochondrial membrane potential, mitochondrial mass, mitochondrial superoxide, glucose-uptake and fatty-acid-uptake assays

Metabolic assays were performed after viability staining, but before tetramer and cell surface staining to minimize impact of T cell activation through TCR stimulation. Cells were stained with the respective dyes in pre-warmed RPMI medium (Life Technologies) and incubated at 37°C for 20 minutes. Mitochondrial membrane potential was measured by Tetramethylrhodamine (TMRM, ThermoFisher Scientific) at a concentration of 5 nM. The mitochondrial mass was measured by MitoTracker™ Deep Red (ThermoFisher Scientific) at a concentration of 5 nM. To measure glucose uptake, cells were incubated with the glucose analog 2-(N-(7-nitrobenz-2-oxa-1,3-diazol-4-yl)amino)-2-deoxyglucose (2-NBDG, Life Technologies) at a concentration of 50 µM. Long-chain fatty acid uptake was measured by staining with 4,4-Difluoro-5,7-Dimethyl-4-Bora-3a,4a-Diaza-*s*-Indacene-3-Hexadecanoic Acid (BODIPY™ FL C_{16}, ThermoFisher Scientific) at a concentration of 30 nM. To measure mitochondrial derived superoxide, cells were incubated with MitoSOX Red (ThermoFisher Scientific) at a concentration of 2.5 µM.

Intracellular staining for metabolic proteins, transcription factors, and histone methylation

All intracellularly stained metabolic proteins except FDFT1 were self-conjugated using the Lightning-Link antibody conjugation kits (Abcam) according to the manufacturer's protocol (**Supplemental Table 4**). After viability staining, cell surface and tetramer staining, cells were permeabilized and fixed for 45 minutes using the eBioscience Foxp3/Transcription Factor Staining Buffer Set (Invitrogen) according to manufacturer's instructions. Following two washes in Permeabilization Buffer, antibodies against metabolic proteins, transcription factors or Histone 3 methylated at Lysine 27 (H3K27me3) were added and cells were incubated for 1 hour at 4 °C. Before analysis, cells were washed two more times in Permeabilization Buffer.

Acquisition and analysis

All flow cytometry samples were acquired on a 5-Laser Aurora Cytek spectral analyzer with SpectroFlo acquisition software (version 3, Cytek). Data were analyzed using FlowJo software (TreeStar) and OMIQ analysis software. A representative gating strategy is shown in Figure S1A. High-dimensional analysis was performed by subsampling on equal numbers of antigen-specific $CD8^+$ T cells followed by UMAP and FlowSOM consensus meta-clustering, Wanderlust trajectory, or clustered heatmap using default settings.

Ex vivo T cell stimulation

PBMCs were cultured in the presence of 9-mer CMV peptide $pp65_{495-503}$ (NLVPMVATV) at a concentration of 10 µg/ml. PBMCs were stimulated with $pp65_{495-503}$ peptide for either 20 hours (short stimulation) to assess intracellular cytokine production, cytokine secretion and functional metabolic changes (e.g., MTDR and TMRM) or 6 days (long stimulation) to assess changes in metabolic protein expression. Because peptide stimulation elicits TCR down-regulation and tetramer internalization but also activation-induced expression of CD137, we used the latter marker for the detection of antigen-reactive $CD8^+$ T cells (1). After long stimulation, the TCR recirculates, and therefore the $pp65_{495-503}$-specific $CD8^+$ T cells were identified by MHC class I tetramers.

For intracellular cytokine staining, cells were stimulated with peptide for 20 hours of which the last 19.5 hours in presence of Brefeldin A (BioLegend, 1 µg/ml). Intracellular cytokine staining was performed as described previously (2). As the relative fraction rather than absolute numbers of cytokine producing CMV-specific $CD8^+$ T cells correlates better with clinical course of CMV infection in transplant patients (3), we calculated the ratio between cytokine producing cells after stimulation (from total $CD8^+$ T cells) and $pp65_{495-503}^+$ cells (from total $CD8^+$ T cells) in unstimulated condition (**Supplemental Figure 1H**). Quantification of cytokine secretion by Legendplex (Biolegend) was performed according to the manufacturer's guidelines.

Metabolic modulators

When assessing the impact of metabolic modulators, reagents were added to the cell culture during stimulation assays for 20 hours or 6 days, respectively. The 78c CD38 inhibitor (CD38i) (Selleckchem) was used at a final concentration of 10 µM. The mitochondrial antioxidants Mitoquinone (Abcam) and MitoTEMPO (Merck Life Sciences) were used at concentrations of 0.05 µM (Mitoquinone) and 5 µM (MitoTEMPO), respectively. 2-DG (Sigma-Aldrich) was used at a concentration of 0.1 µM, etomoxir (Abcam) at a concentration of 200 µM, oligomycin (Abcam) at a concentration of 0.5 µM, SRT1720 (Selleckchem) at a concentration of 0.5 µM, Ex527

(Selleckchem) at a concentration of 50 µM and Nicotinamide mononucleotide (Sigma-Aldrich) at a concentration of 100 µM.

SCENITH

PBMCs were directly subjected to SCENITH (**Figure 3J; Supplemental Figure 3I**) or PBMCs were first stimulated with $pp65_{495\text{-}503}$ peptide (NLVPMVATV) for 4 hours followed by SCENITH (**Figure 4J; Supplemental Figure 4E**). In case of CD38 inhibition, PBMCs were pre-incubated for 16 hours with CD38i or vehicle, and then directly subjected to SCENITH (**Supplemental Figure 7E**) or first stimulated for 4 hours with $pp65_{495\text{-}503}$ peptide (**Figure 4L**). SCENITH was performed in accordance with the original SCENITH protocol (4). Briefly, cells underwent a 20-minute treatment with either 100 mM 2-DG, 1 µM oligomycin (O), or a sequential combination of both drugs at 37 °C, 5% CO_2. Subsequently, puromycin (10 µg/mL) was added to the culture for additional 20 minutes. Afterwards, cells were washed and flow cytometry staining was performed. Cells were fixed and permeabilized using the eBioscience Foxp3/Transcription Factor Staining Buffer Set (Invitrogen) and stained intracellularly with the monoclonal anti-puromycin antibody for 45 min. Values for glucose dependence, mitochondrial dependence, glycolytic capacity, and FAO and AAO capacity were calculated.

Nanostring nCounter Gene expression analysis

PBMCs were thawed and rested overnight in IMDM. The next day, PBMCs were labelled with Zombie NIR to exclude dead cells, and anti-CD8 mAbs plus $pp65_{495\text{-}503}$ (NLVPMVATV)/tetramers. Subsequently, pp65-specific $CD8^+$ T cells (17000 cells/sample) were sorted on a Cytoflex SRT (Beckman Coulter) and collected in cold IMDM (2% FCS) (**Figure S2A**). Next, cells were washed three times in PBS and lysed in buffer containing 1 portion of RLT buffer (Qiagen) and 2 portions of UltraPure™ DNAse/RNase-free distilled water (Invitrogen). Lysates were immediately placed on dry ice and subsequently stored at -80 °C until the day of analysis. Samples were analyzed in the NanoString® nCounter FLEX platform according to manufacturer's instructions. The nCounter® Metabolic Pathways Panel (768 genes) was used to interrogate the metabolic profile. Briefly, 5 µl of cell lysate was mixed with reporter probes, capture probes and hybridization buffer, and proteinase K (0.45 mg/ml, Thermo Fisher Scientific), and hybridized at 65°C for 17 hours. Samples were then processed on the NanoString Prep station and cartridges were read on the NanoString Digital Analyzer. RNA count data were normalized, scores for different pathways were calculated, automated pathway analysis based on the expression of predefined genes and differential expression analysis were performed using the nSolver™ software (version 4.0) including the advanced analysis package (version 2.0.134). Volcano plot was created with GraphPad Prism (version 8, La Jolla, CA, United States). Heatmaps were created with Morpheus, https://software.broadinstitute.org/morpheus. Protein-protein interaction analysis and pathway elucidation analysis were performed using STRING (5), with a minimum required interaction score of 0.7 (high confidence).

In vivo viral infection and immunosuppressive medication

C57BL/6 mice were i.p. infected with murine cytomegalovirus (MCMV-Smith, obtained from the American Type Culture Collection, ATCC VR-194; Manassas, VA) (2×10^4 PFU). Mice receiving immunosuppressive treatment were injected on day 3 post-infection with tacrolimus (3 mg/kg, s.c.) and dexamethasone (1 mg/kg, i.p.), and subsequently every 24 hours until day 7, and then every 48 hours until sacrifice. Mice were weighed 3 times per week, but no weight loss or other

signs of discomfort were observed. On day 20 after MCMV infection mice were sacrificed. Single cell suspensions from the spleen were prepared by mincing the tissue through a 70 µm cell strainer. Contaminating erythrocytes were removed by ammonium chloride buffer. Salivary glands were collected from euthanized mice and snap-frozen on dry ice. Samples were stored at -80°C until the viral load was determined by quantitative real-time PCR as described previously (6).

In vivo treatment with CD38i (78c)

C57BL/6 mice were infected with MCMV-Smith and received immunosuppressive treatment as described above (until sacrifice). Mice were weighed 3 times per week. On day 20 after MCMV infection the treatment group received 15 mg/kg/dose 78c twice daily i.p. for 7 days as described previously (7). Control mice received vehicle (5% DMSO, 40% PEG300, 5% Tween 80 and 50% NaCl 0.9%). On day 27 after infection mice were sacrificed. Single cell suspensions from the spleen were prepared by mincing the tissue through a 70 µm cell strainer. Contaminating erythrocytes were removed by ammonium chloride buffer. Intracellular cytokine staining was performed after 5 hours stimulation in presence of short M38 peptide (SSPPMFRV) and Brefeldin A as described previously (6). Livers were collected from euthanized mice and snap-frozen on dry ice. Samples were stored at -80°C until the viral load was determined by quantitative real-time PCR as described previously (6).

In vivo viral infection and mass cytometry analysis

Expression data of PD-1, CD38 and CD39 on viral-specific $CD8^+$ T cells elicited by different types of infection (i.e., LCMV-Armstrong, LCMV-clone 13, MCMV-*ie2*-GP33, MCMV-*ie2*-E7, MCMV-*ie2*-FKBP-E7) were derived from our previous study by van der Gracht et al. (8). Briefly, mice were infected with the aforementioned viruses and at day 50 post infection the viral-specific $CD8^+$ T cell responses were characterized by mass cytometry.

References

1. Wolfl M, et al. Activation-induced expression of CD137 permits detection, isolation, and expansion of the full repertoire of CD8+ T cells responding to antigen without requiring knowledge of epitope specificities. *Blood.* 2007;110(1):201-10.
2. Pardieck IN, et al. A third vaccination with a single T cell epitope confers protection in a murine model of SARS-CoV-2 infection. *Nat Commun.* 2022;13(1):3966.
3. Benz C,et al. Activated virus-specific T cells are early indicators of anti-CMV immune reactions in liver transplant patients. *Gastroenterology.* 2002;122(5):1201-15.
4. Argüello RJ, , et al. SCENITH: A Flow Cytometry-Based Method to Functionally Profile Energy Metabolism with Single-Cell Resolution. *Cell Metab.* 2020;32(6):1063-75.e7.
5. Szklarczyk D, et al. The STRING database in 2023: protein-protein association networks and functional enrichment analyses for any sequenced genome of interest. *Nucleic Acids Res.* 2023;51(D1):D638-d46.
6. Redeker A, Welten SP, Arens R. Viral inoculum dose impacts memory T-cell inflation. *Eur J Immunol.* 2014;44(4):1046-57.
7. Tarragó MG, et al. A Potent and Specific CD38 Inhibitor Ameliorates Age-Related Metabolic Dysfunction by Reversing Tissue NAD(+) Decline. *Cell Metab.* 2018;27(5):1081-95.e10.
8. van der Gracht ETI, et al. Memory CD8(+) T cell heterogeneity is primarily driven by pathogen-specific cues and additionally shaped by the tissue environment. *iScience.* 2021;24(1):101954.

Supplementary Tables

Supplemental Table 1: Main study population

	Controllers	Non-controllers	Healthy controls
N	22	14	13
Age (years)	51 (28-78	49.5 (21-70)	55 (31-79)
Sex	60% male	58% male	54% male
eGFR (ml/min/1.73 m^2)	49.9 (16.8-107.3)	47.8 (17.0-105.3)	91.2 (76-115)
Serum creatinine (mg/dl)	1.45 (0.77-3.38)	1.52 (0.72-3.45)	1.01 (0.56-1.21)
Time between transplantation and blood sampling (months)	15 (4-30)	13 (3-32)	n.a.
CMV risk-constellation	22.7% high-risk<br>77.3% intermediate-risk	42.8% high-risk<br>57.2% intermediate-risk	n.a.
Detectable CMV-replication (in blood) in the past	54.5%	100%	n.a.
Time between latest CMV-replication onset and blood sampling (days)	65 (12-132)<br>*in case of detectable replication*	45 (18-72)	n.a.
Viral load in blood[A] (IU/ml)	3 patients with detectable replication (70; 76; 93)	4796 (367-34552)	n.a.
Highest ever recorded viral load in blood[B] (IU/ml)	98 (66-280)<br>*in case of detectable replication*	28137 (1251-166299)	n.a.
Previous CMV disease (organ invasive disease)	0 %	28.5%	n.a.
Treatment with valganciclovir[A]	18% (*prophylactic*)	50% (*prophylactic and therapeutic*)	n.a.
Treatment with valganciclovir[B]	31.2 % (*prophylactic*)	100 % (*prophylactic and therapeutic*)	n.a.
Treatment with anti-CMV immunoglobulin[A]	0 %	14%	n.a.
Immunosuppressive medication[A]			
tacrolimus	91%	93%	n.a.
mycophenolate	86%	86%	n.a.
prednisone	100%	100%	n.a.
everolimus	9%	14%	n.a.

[A]at time of blood sampling; [B]present or in the past; n.a. = not applicable. Numbers are given as mean and range for metric variables and percentage for categorical variables.

Supplemental Table 2: In- and exclusion criteria

Inclusion criteria	Exclusion criteria
CMV high- or intermediate-risk (KTR), anti-CMV IgG positive (HC)	Rejection therapy within last 3 months
HLA-A*02 positive	eGFR <15ml/min/m^2
At least two mRNA vaccinations against SARS-CoV-2 (between 14 days and 6 months before blood sampling)	Acute SARS-CoV-2-infection (positive PCR within 14 days before blood sampling)
Last kidney transplantation between 3 and 36 months ago	<40 virus-specific $CD8^+$ T cells in flow cytometric analysis

Supplemental Table 3: MHC class I tetramers

MHC class I Tetramer	Fluorophore	Peptide	Supplier
HLA-A*02 tetramer HCMV pp65$_{495-503}$	PE	NLVPMVATV	LUMC tetramer facility
HLA-A*02 tetramer HCMV pp65$_{495-503}$	APC	NLVPMVATV	LUMC tetramer facility
HLA-A*02 tetramer SARS-CoV-2 Spike$_{269-277}$	PE	YLQPRTFLL	LUMC tetramer facility
HLA-A*02 tetramer SARS-CoV-2 Spike$_{269-277}$	APC	YLQPRTFLL	LUMC tetramer facility
HLA-A*02 tetramer HCMV IE-1$_{316-324}$	PE	VLEETSVML	LUMC tetramer facility
H2-Kb tetramer MCMV M38$_{316-323}$	APC	SSPPMFRV	LUMC tetramer facility

Supplemental Table 4: Antibodies and cell viability probes

Antibody	Fluorophore	Clone	Supplier	Catalogue number
CD3	V450	UCHT1	BD Biosciences	560365
CD3	BUV395	UCHT1	BD Biosciences	563548
CD8a	BUV805	SK1	BD Biosciences	612889
CD8a	BV785	SK1	BioLegend	344739
CD45RA	BUV496	HI100	BD Biosciences	750258
CCR7	BV785	G043H7	BioLegend	353230
CD38	APC-fire810	HIT2	BioLegend	303549
CD57	BV605	QA17A04	BioLegend	393303
KLRG1	PE-Cy7	14C2A07	BioLegend	368613
PD-1	BUV615	EH12.1	BD Biosciences	612991
CD25	BV711	M-hla	BioLegend	356138
CD137	BV650	4B4-1	BioLegend	309828
CD98	BUV395	UM7F8 (RUO)	BD Biosciences	744508
CD69	PE-Cy7	FN50	Biolegend	310912
CD8a	BUV395	53-6.7	BD Biosciences	565968
CD44	BUV805	IM7	BD Biosciences	741921
KLRG1	BV785	2F1	Biolegend	138429
CD62L	MEL-14	PE-Dazzle594	Biolegend	104408
CD38	90	BUV737	BD Biosciences	741748
IFN-y	XMG1.2	APC	Thermo Fisher	17-7311-82
TNF	MP6-XT22	FITC	Biolegend	506301
GLUT1	BSA/Azide free DyLight 405	EPR3915	Abcam	ab252403 ab201798
ACC1	BSA/Azide free PE-Cy7	EPR23235-147	Abcam	ab272704 ab102903
CPT1a	BSA/Azide free PE-Cy5	EPR21843-71-2F	Abcam	ab235841 ab102893
Cytochrome C	BSA/Azide free PE-Cy7	7H8.2C12	Abcam	ab237966 ab102903
SDHA	BSA/Azide free Alexa Fluor 647	EPR9043(B)	Abcam	ab240098 ab269823
G6PD	BSA/Azide free APC-Cy 7	EPR20668	Abcam	ab231828 ab102859
PKM	BSA/Azide free PE	EPR10138(B)	Abcam	ab206129 ab102918
ATP5a	BSA/Azide free DyLight 488	EPR13030(B)	Abcam	ab231692 ab201799
MCAD	BSA/Azide free	EPR3708	Abcam	ab239914

	DyLight 405			ab201798
ATGL	BSA/Azide free<br>Alexa Fluor 647	EPR19650	Abcam	ab240381<br>ab269823
FDFT1	DyLight 550	OTI2F10	Novus Biologicals	NBP2-70715R
FASN	BSA/Azide free<br>APC-Cy7	EPR7466	Abcam	ab221934<br>ab102859
Citrate-<br>Synthase	BSA/Azide free<br>DyLight 488	EPR8067	Abcam	ab233838<br>ab201799
ALDOA	Alexa Fluor 647	EPR23181-39	Abcam	ab275162
Puromycin	Alexa Fluor 647	2A4	Biolegend	381507
FoxP3	RB780	259D/C7	BD Biosciences	568682
EOMES	PE-eFluor 610	WD1928	Invitrogen	61-4877-41
T-BET	Kiravia Blue 520	4B10	BioLegend	644837
IRF-4	PE-Cy7	IRF4.3E4	Biolegend	646413
BLIMP-1	PE	6D3	BD Biosciences	564702
PGC-1α	PE	Polyclonal	Novus Biologicals	NBP1-<br>04676PE
Tri-Methyl-<br>Histone H3<br>(Lys27)	Alexa Fluor 647	C36B11	Cell Signaling<br>Technology	12158

**Cell viability probe**	**Supplier**	**Catalogue number**
Zombie NIR	BioLegend	423106
Live-Dead Fixable Blue	Thermo Fisher	L23105

Supplemental Table 5: Spike-specific HLA class I tetramer mix

HLA type	Protein	Peptide	Fluorochrome
HLA-A*01:01	Spike	LTDEMIAQY	PE
HLA-A*02:01	Spike	FIAGLIAIV	PE
HLA-A*02:01	Spike	FLPFFSNV	PE
HLA-A*02:01	Spike	RLNEVAKNL	PE
HLA-A*02:01	Spike	RLQSLQTYV	PE
HLA-A*02:01	Spike	VLNDILSRL	PE
HLA-A*02:01	Spike	VVFLHVTYV	PE
HLA-A*02:01	Spike	YLQPRTFLL	PE
HLA-A*03:01	Spike	GTHWFVTQR	PE
HLA-A*03:01	Spike	KCYGVSPTK	PE
HLA-A*11:01	Spike	GTHWFVTQR	PE
HLA-A*11:01	Spike	KCYGVSPTK	PE
HLA-A*24:02	Spike	IYKTPPIKDF	PE
HLA-A*24:02	Spike	QYIKWPWYI	PE
HLA-A*24:02	Spike	RFDNPVLPF	PE
HLA-A*24:02	Spike	TQDLFLPFF	PE
HLA-A*24:02	Spike	TYVPAQEKNFT	PE
HLA-B*07:02	Spike	LPQGFSAL	PE
HLA-B*07:02	Spike	MIAQYTSAL	PE
HLA-B*07:02	Spike	SPRRARSVA	PE
HLA-B*15:01	Spike	CVADYSVLY	PE
HLA-B*15:01	Spike	LVKNKCVNF	PE
HLA-B*15:01	Spike	VASQSIIAY	PE
HLA-B*35:01	Spike	FVSNGTHWF	PE
HLA-B*35:01	Spike	QPTESIVRF	PE
HLA-B*35:01	Spike	VASQSIIAY	PE
